# Nontargeted Urinary Profiling Strategy for Endocrine-Disrupting Chemicals in Women with Ovarian Malignancies

**DOI:** 10.1021/acs.est.4c13290

**Published:** 2025-04-22

**Authors:** Helena Plešnik, Žan Rekar, Stefanela Stevanović, Irma Virant-Klun, Senka Imamović Kumalić, Mateja Sladič, Darja Mazej, Janja Snoj Tratnik, Milena Horvat, Tina Kosjek

**Affiliations:** † Department of Environmental Sciences, Jožef Stefan Institute, 1000 Ljubljana, Slovenia; ‡ Jožef Stefan International Postgraduate School, 1000 Ljubljana, Slovenia; § Faculty of Computer and Information Science, University of Ljubljana, 1000 Ljubljana, Slovenia; ∥ Clinical Research Centre, University Medical Centre Ljubljana, 1000 Ljubljana, Slovenia; ⊥ Faculty of Medicine, University of Ljubljana, 1000 Ljubljana, Slovenia; # Division of Obstetrics and Gynecology, University Medical Centre Ljubljana, 1000 Ljubljana, Slovenia

**Keywords:** ovarian cancer, biomarkers, exposure, nontargeted screening, high-resolution mass spectrometry, analytical coverage, reversed-phase, HILIC

## Abstract

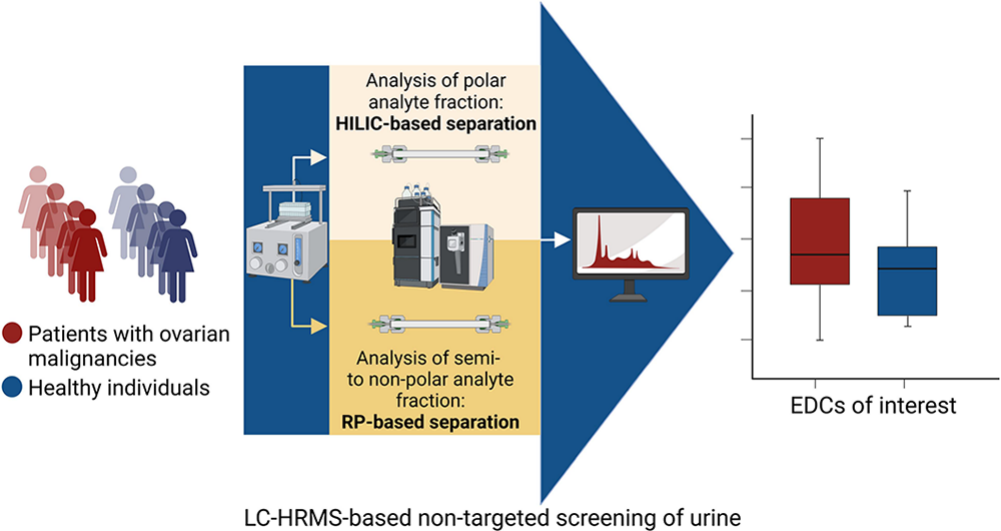

Endocrine-disrupting chemicals (EDCs), including known and unknown parent compounds, their metabolites, and transformation products, are pervasive in daily life, posing increasing risks to human health and the environment. This study employed a high-resolution mass spectrometry-based nontargeted screening approach, integrating polar (HILIC) and reversed-phase separations to expand the chemical space coverage and, supported by open-science tools and resources, evaluated urinary chemical profiles to assess internal EDC exposure. Among 106 annotated biomarkers of exposure, six exhibited significantly higher normalized intensities in patients with ovarian malignancies compared to healthy controls (*p* < 0.05). This suggests their greater exposure to phthalates (diethylhexyl phthalate and diethyl phthalate), pesticides (metolachlor metabolite and 4-nitrophenol), a UV filter (benzophenone-1), and an industrial byproduct (4-methyl-2-nitrophenol). These compounds may interfere with hormonal regulation, potentially contributing to cancer development. While these findings highlight potential differences in internal EDC exposure, the study primarily demonstrates the applicability of nontargeted urinary profiling for chemical exposure assessment. By providing new insights into EDCs burden and its pathological implications, this work contributes to advancing next-generation chemical risk assessment within the European Partnership for the Assessment of Risks from Chemicals initiative and supports the development of preventive strategies to mitigate environmental cancer risks.

## Abstract 2

This research highlights how specific environmental contaminants, such as phthalates and pesticides, which may be linked to ovarian malignancies, support the need for preventive strategies addressing environmental health impacts.

## Introduction

Ovarian cancer (OC) remains one of the most lethal gynecological malignancies worldwide. Despite advances in diagnostic technologies and treatments, the 5-year survival rate for advanced-stage OC remains around 50%.^1^ Risk factors for OC include genetic predispositions, reproductive history, hormonal imbalances, and lifestyle behaviors.^2^ Environmental exposures, including endocrine-disrupting chemicals (EDCs), have gained significant attention as potential contributors to hormone-sensitive cancers, such as OC, with an estimated 90% of cancers attributed to environmental factors.^3^ EDCs, a heterogeneous group of compounds, interfere with hormonal systems by mimicking, blocking, or altering hormone function. Although research has extensively examined the effects of EDCs on breast, prostate, and testicular cancers, their role in OC remains underexplored.^4^ Emerging evidence links exposure to compounds such as phthalates,^5^ bisphenols,^6,7^ triclosan,^8^ and chlorotriazines^9^ with increased OC risk. Additionally, in vitro studies suggest that also benzophenones, such as benzophenone-1 (BP-1), may stimulate OC cell proliferation,^10^ likely through mechanisms involving oxidative stress, chronic inflammation, and disruption of hormonal homeostasis. These findings underscore the need for a deeper understanding of EDCs’ role in OC etiology, particularly in the context of cumulative and lifelong exposures. Borderline ovarian tumors (BOT), which are less aggressive and carry a better prognosis than OC, share some overlapping risk factors. However, their association with environmental exposures, including EDCs, remains poorly understood.^11^ Investigating both OC and BOT within the exposome framework – encompassing the entirety of an individual’s environmental exposures from conception onward^12^ – provides a comprehensive approach to elucidate the role of environmental factors in gynecological cancers. In this sense, the exposome concept offers a more holistic understanding of environmental contributions to cancer risk, and along with the technological progress and innovation, new methodological approaches have emerged that enable us to expand our knowledge about environmental and human chemical exposure.^13^

Urinary biomonitoring has emerged as a powerful method for assessing environmental exposures due to its non-invasive nature and ability to reflect both endogenous and exogenous compounds. Despite these advantages, current methodologies face significant challenges in detecting low-abundance contaminants, particularly in nontargeted and suspect screening (NTS/SS) approaches. Limitations include incomplete chemical coverage, particularly of highly polar biomarkers of exposure (BoEs),^14^ and the complexity of identifying low-concentration analytes against a backdrop of endogenous compounds.

This study explores chemical exposure profiles in women with ovarian malignancies (OM), focusing on EDCs and their potential role in OC. Advanced NTS/SS methodologies integrated with human biomonitoring aim to address critical gaps in exposure science. A novel urine sample preparation method was developed for dual-column ultrahigh-performance liquid chromatography (UHPLC), combining hydrophilic interaction liquid chromatography (HILIC) and reversed-phase (RP) separations with HRMS. This approach enhances compound detection across a wide polarity range, improving the detection and identification of urinary BoEs. Applied to samples from OM patients and healthy controls (HC), the approach identifies exposure differences and their potential links to cancer risk. The study advances analytical methods while providing insights into environmental contributions to cancer. Findings support regulatory frameworks for chemical safety and align with the goals of the Partnership for the Assessment of Risks from Chemicals (PARC) initiative.^15^ Particularly, it focuses on early warning tools for chemicals of emerging concern.

## Materials and Methods

Materials and Chemicals are listed in S1 (Chapter 1) and reference standards are listed in Table S2-A1.

### Samples

First-morning urine samples involved those of 25 healthy pregnant women (healthy controls, HC, average age 33.6 ± 2.8) with a singleton pregnancy between 37 and 41 weeks of gestation and without chronic diseases, and 25 women diagnosed with OM (average age 46.4 ± 16.1), of which 10 with BOT (average age 40.3 ± 16.1) and 15 with OC (average age 50.5 ± 14.7), all of whom provided informed consent. The diagnosis of BOT and OC was based on the histopathological examination. All participants provided first-morning urine samples after fasting.

Urine samples were collected in polypropylene containers prewashed with 10% nitric acid, aliquoted, and immediately stored at −80 °C to preserve sample integrity. Shortly prior to analysis, samples were thawed at room temperature. The study was conducted in accordance with the Declaration of Helsinki and was approved by the Slovenian National Commission for Medical Ethics (license no.: 0120-158/2022/9).

For method development, validation, and column conditioning throughout the study, pooled urine samples from 5 healthy donors were used. Procedural bank samples used LC-MS grade water as the matrix.

### Sample Preparation

The protocol was adopted from our previous studies.^16,17^ To 1 mL of thawed urine, 430 μL of LC-MS grade water, 50 μL of 3 M sodium acetate buffer (pH 5.2), and 20 μL of β-glucuronidase/arylsulfatase solution (*Helix pomatia*, type H-2) were added to achieve the final enzyme concentration of 135 U/mL. The mixture was incubated at 37 °C for 16 h on an orbital thermo-shaker to facilitate deconjugation. For solid-phase extraction (SPE), we conditioned a 60 mg sorbent packed in an Oasis-HLB 96-well plate with 1 mL of ethyl acetate (EtOAc), 1 mL of methanol (MeOH), and equilibrated it with 1 mL of LC-MS grade water. After loading the sample, we eluted the analytes from the sorbent with 1.5 mL of 30% (v/v) MeOH in water (the “wash fraction”) and collected it separately from the subsequent 1.5 mL of 10% (v/v) EtOAc in acetonitrile (ACN) (“elution fraction”). We added isotopically labeled internal standards (IS) (listed in Table S2-A1) to both fractions at final concentrations of 500 μg/L in the wash fraction and 100 μg/L in the elution fraction. These concentrations were optimized based on the different instrumental methods used and were necessary due to differences in the sensitivity and ionization efficiency of the methods. 10 μL of DMSO were added exclusively to the elution fraction to serve as a keeper, with the volume chosen to effectively minimize analyte loss while preventing interference with LC analysis. Both fractions (wash and elution) were then dried under a gentle nitrogen stream while being heated to 40 °C in heat blocks to speed up the evaporation. Prior to the instrumental analysis, we reconstituted the wash and elution fractions with 250 μL of 80% (v/v) ACN in water and 250 μL of 10% (v/v) ACN in water, respectively, and filtered samples using 0.2 μm syringe filters with regenerated cellulose membrane. Procedural blank samples, calibrators, and all quality control (QC) standards, except for system suitability controls, were prepared following the same procedure.

### Instrumental Analysis

Instrumental analyses were performed using a Thermo Fisher Vanquish UHPLC system coupled to a Thermo Fisher Orbitrap Exploris 240 mass spectrometer (Thermo Fisher Scientific Inc., Waltham, MA, USA), which was calibrated accordingly with the manufacturer’s instructions. Scan-to-scan mass correction was applied during acquisition to ensure the highest mass accuracy throughout the analysis. HILIC and RP-based acquisitions were applied to the wash and elution fractions, respectively. In the HILIC-based acquisition, we achieved the separation using a Waters BEH amide (2.1 × 100 mm, 1.7 μm) column with (A) ACN and (B) 20 mM ammonium formate with 0.1% formic acid (pH 3.5) as mobile phases. The elution gradient was as follows: 5% B (0–2 min), 5–45% B (2–15 min), 45% B (15–16 min) 45–5% B (16–16.5 min), 5% B (16.5–30 min). The flow rate was 0.4 mL/min, the column temperature was 35 °C and the injection volume was 5 μL. Heated electrospray ionization (HESI) was used as the ionization source with the following settings: capillary voltage, 3.5 and −2.5 kV for positive and negative mode, respectively; sheath gas, 50 AU; auxiliary gas, 10 AU; sweep gas, 1 AU; ion transfer tube temperature, 325 °C; vaporizer temperature, 350 °C. The full scan mass acquisition scanned the mass range of 80–1000 *m*/*z* at the resolution of 90,000 fwhm, while the MS2 data was acquired at the resolution of 30,000. The centroiding was performed directly, without postacquisition conversion. The data-dependent acquisition (DDA) involved intensity threshold for the MS/MS events set to 1.0 × 10^3^, with the top 8 most intense ions selected for fragmentation. Dynamic exclusion with a 9 s time window and 5 ppm mass tolerance was applied. A 20% apex window was used for DDA, with an isolation window of 1.5 *m*/*z* for precursor ions. Higher-energy collisional dissociation (HCD) was applied with normalized stepped collision energies of 30, 50, and 150% to ensure comprehensive fragmentation.

In the RP-based acquisition, we used a Waters Acquity HSS-T3 (2.1 × 100 mm, 1.8 μm) column with (A) ACN with 0.1% formic acid and (B) water containing 0.1% formic acid (pH 2.6) as the mobile phases. The elution gradient was as follows: 95–75% B (0–3 min), 75–60% B (3–7 min), 60–40% B (7–15 min), 40–30% B (15–18 min), 30–0% (18–24.5 min), 0% B (24.5–26.5 min), 0–95% B (26.5–27.5 min), 95% B (27.5–33 min). The flow rate was 0.3 mL/min, the column temperature was 35 °C and the injection volume was 5 μL. The HESI settings involved capillary voltage of 4.5 kV for positive and −3.0 kV for negative ionization mode; sheath gas, 40 AU; auxiliary gas, 15 AU; sweep gas, 2 AU; ion transfer tube temperature, 350 °C; vaporizer temperature, 400 °C. The full scan mass acquisition covered the mass range of 100–900 *m*/*z* and was acquired with a resolution of 90,000 while MS2 data was acquired with a resolution of 30,000. The centroiding was performed directly, without postacquisition conversion. The DDA intensity threshold for the MS/MS events was set to 1.0 × 10^4^, and the top 7 most intense ions were selected for fragmentation. Dynamic exclusion with a 9 s time window and 5 ppm mass tolerance was applied. A 20% apex window was used for DDA, with an isolation window of 1.5 *m*/*z* for precursor ions. HCD was applied with normalized stepped collision energies of 30, 50, and 150% to ensure comprehensive fragmentation.

### Validation, Quality Assurance, and Quality Control

Validation followed a modified protocol from Grijseels et al.,^18^ assessing mass accuracy, retention time (RT) variability, method repeatability, trueness, linearity, and limit of detection (LOD) of the reference standards listed in S2-A1.^18^ Validation parameters and the set criteria are shown in Table 1.

**Table 1:** Outline of the Validation Plan for the Analytical Method, Including Specified Acceptance Limits for Mass Accuracy, RT Variability, Method Repeatability, Trueness, Linearity, LOD, and Carry-Over^a^

parameter	definition	criteria
mass accuracy	difference between measured and theoretical *m*/*z*	mass error < 5 ppm
RT variability	RT of QC standards compared to the defined RT	RP: RT error < 0.10 min
		HILIC: RT error < 0.25 min
method repeatability	% CV of peak heights, normalized with IS (3 QC standards, spiked with 100 μg/L)	< 30% CV
trueness	% difference between spiked and experimentally determined concentration, expressed as % Bias (3 QC standards, spiked with 100 μg/L)	< ±35% bias
linearity	pooled urine samples, spiked with reference standards at 1, 5, 25, 50, 100, and 150 μg/L injected to establish linearity of response and detection range.	evaluated, but not used as exclusion criteria
LOD	lowest calibrator with a signal/noise ratio ≥ 3	evaluated, but not used as exclusion criteria
carry-over	solvent blanks injected after the highest calibrator	< 20% of the LOD peak height for spiked reference standard

a CV: coefficient of variation; HILIC: hydrophilic interaction liquid chromatography; IS: internal standard; LOD: limit of detection; ppm: parts per million; QC: quality control; RP: reversed-phase; RT: retention time.

We prepared two separate batches, one for validation and one for sample analysis. The latter was randomized to ensure unbiased distribution and robust results. We started each batch with a reconstitution solvent as a system blank. System control standards were solvents spiked with reference standards at 150 and 300 μg/L, which we injected at the beginning and end of each batch to ensure consistent system performance, evaluate mass accuracy (mass error < 5 ppm) and RT variation (±0.25 min (HILIC) and ± 0.10 min (RP)) of the spiked reference standards. Three quality control (QC) standards were prepared from pooled urine spiked with reference standards at 100 μg/L pre-extraction. Each QC was injected twice per batch, resulting in a total of 6 QC injections total, performed every 10 samples to monitor system stability. The IS signals were monitored in all samples and QCs to confirm proper injection and identify any anomalies. The reference standard and IS lists are provided in Table S2-A1, and monitored system control reference standards are listed in Table S2-A2. The QC charts were established to control for any variations across the batches and are presented in S1 (Figures S1-1–4).

### Data Analysis

Raw data were processed using mzmine 4.3.0,^19^ with processing parameters detailed in S1-2.1 and S1-2.2. Features present in the procedural blanks (ratio between samples to procedural blanks < 3), those without MS2 data, and features eluted in the void were excluded. Feature heights were normalized using the closest eluting IS (normalized intensities). Heights were preferred over peak areas to minimize inaccuracies in baseline estimation, particularly for low abundance analytes, a common challenge in NTS data preprocessing.

**Figure 1:**
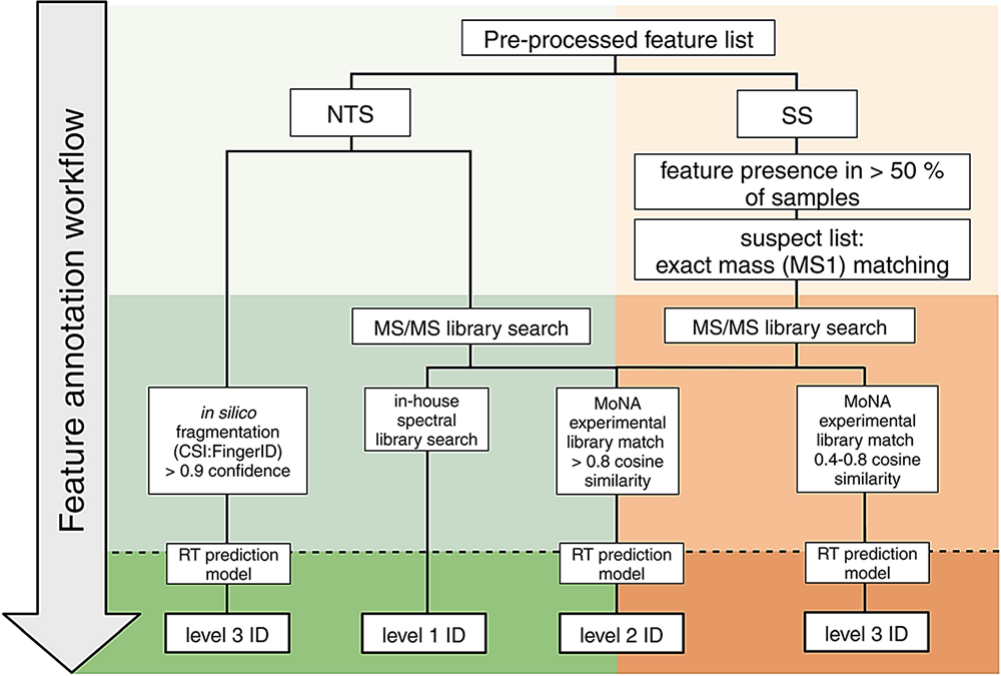
Feature annotation workflow.

*BoEs annotation* followed two approaches as illustrated in Figure 1 and was based on a modified version of the Schymanski scale.^20^*Nontargeted screening* involved matching MS2 spectra to libraries. Matches to the in-house library (positive.mgf and negative.mgf), which was built using our reference standards, complying with our established criteria (mass error of the parent ion < 5 ppm, three matched MS2 signals (mass error < 10 ppm), and RT variation ± 0.25 min (HILIC) and ± 0.10 min (RP)), were assigned level 1 ID confidence. Matches to the MoNA (Mass Bank of North America) experimental MS2 library,^21^ applying the same mass accuracy criteria as level 1, along with cosine similarity > 0.8, were reported as level 2 ID confidence. In silico fragmentation and identification were performed by uploading MS2 spectra to SIRIUS+CSI:FingerID,^22^ and then generating possible structural candidates from selected libraries (KEGG,^23^ ChEBI,^24^ HMDB,^25^ and GNPS^26^). The candidates with match confidence > 0.9 and with all matching substructures were reported at the level 3 ID confidence. Urine endogenous and food-derived biomarkers were filtered out at this stage using information from HMDB.^25^

*Suspect screening* annotation approach was applied to features present in more than 50% of samples. It involved matching MS1 data to an in-house suspect list with a mass tolerance of 5 ppm, followed by MS2 data matching to the MoNA experimental library. The suspect list included 801 entries with name, monoisotopic mass, *m*/*z* values, IUPAC name, InChI, and canonical SMILES for molecular ions of xenobiotic compounds compiled from Exposome Explorer,^27^ T3DB,^28^ and HBM4 EU CECscreen.^29^ The inclusion list contained entries of various classes of contaminants, including pesticides, plasticizers, plastic-related chemicals, personal care products, and persistent organic pollutants. Matches with similarity scores above 0.8, were reported as level 2 ID annotations, and matches with similarity scores from 0.4 to 0.8, considering only the best match, were reported as level 3 ID annotations.

Finally, RT prediction models, developed for each method using the Retip package,^30^ were applied to exclude tentatively annotated compounds whose RT deviated by more than three times the mean absolute error of the corresponding model.

For statistical analysis focusing on BoEs, pharmaceutical residues, and caffeine were excluded, as their presence primarily reflects voluntary use or lifestyle choices rather than background exposure. The analysis was conducted based on the annotated BoEs to assess differences between (a) HC and OM, and (b) HC, BOT, and OC groups separately. Urine dilution was corrected using specific gravity adjustment prior to analysis. A detailed description of the procedure and calculation is provided in S1 (Chapter 5).

a) Normality of the data was assessed using the Shapiro-Wilk test, and homoscedasticity across the groups was evaluated using Levene’s test. Based on the results, a nonparametric Mann–Whitney U-test was selected to compare the normalized intensities of BoEs between the two independent groups (*p* < 0.05).
b) Variations in distribution among the three groups (HC, BOT, and OC), as well as trends in median values across the health spectrum were assessed solely for the BoEs exhibiting significant differences (*p* < 0.05) in step (a), with the objective to link higher median normalized intensities with the condition severity. For those BoEs identified in step (a), a Kruskal–Wallis test was applied to the three groups, followed by pairwise Mann–Whitney U-tests for each group pair (HC vs BOT, HC vs OC, and BOT vs OC) to allow comparisons of distributions among the specific group combinations. For the pairwise comparisons, the Bonferroni correction was applied to adjust for multiple testing, ensuring that the significance level was maintained across all group combinations. Therefore, a threshold of *p* < 0.017 was set as the criterion for statistical significance.

To ensure thorough reporting of the research, the NTA Study Reporting Tool (SRT)^31,32^ was used in the preparation of this manuscript.

## Results and Discussion

### Analytical Method Development

The analytical method was optimized to maximize compound coverage while minimizing sample volume and simplifying preparation to reduce processing time and contamination risk. A sequential wash and elution protocol was used, where the wash fraction with highly polar compounds was analyzed with HILIC-based approach and the elution fraction, containing semipolar compounds, was analyzed with RP-based approach. The selection of analyte sets was purpose-driven, with 57 compounds for method development, 90 for detection rate assessment, and 32 for QA/QC monitoring, ensuring a balance between comprehensive evaluation and practicality (Table S2-A1).

To determine the optimal solvent composition, 57 representative reference standards spanning diverse BoEs and a broad XLogP3 range (−7.5 to 7.4) were evaluated (Table S2-A1 and Figure S1-5). Testing various solvent compositions revealed an optimal balance between the two complementary fractions, maximizing compound abundance and coverage across positive and negative ionization modes in both HILIC and RP approaches.

Following scaling the reference standards’ normalized intensities from 0 to 1, the outcomes are illustrated as heatmaps for HILIC (Figure S1-6) and RP (Figure S1-7). Notably, the wash fraction demonstrated a higher abundance of polar compounds, with some (e.g., malathion dicarboxylic acid, bleomycin) being exclusively present there (Figure S1-6). Conversely, the elution fraction exhibited the highest presence of less polar compounds, as evident from the predominant red color in the top left corner in Figure S1-7, particularly when using the stronger elution solvent, E2 (30% EtOAc in ACN).

To quantify the overall efficiency of different solvent combinations, scaled normalized efficiencies were summed as presented in Figure S1-8. While differences were minor, the optimal combination was identified as 30% MeOH in water as the wash solvent and 10% EtOAc in ACN as the elution solvent.

### Analytical Method Performance Assessment

We evaluated the analytical method’s performance by assessing mass accuracy, RT variability, method repeatability, trueness, linearity, and LOD on a selected subset of reference standards for positive and negative acquisition modes in HILIC and RP-based instrumental approaches. The summarized results are presented in Table S2-A3 and confirm linear responses within the tested concentration ranges, with most *R*^2^ values exceeding 0.900. While a few exceptions showed slightly lower linearity, the lowest *R*^2^ observed was 0.818 in the case of 5-fluorouracil, which is still within a reasonable range. The demonstrated LODs for many compounds are substantially low, especially with the RP-based approach, enabling robust detection at trace levels, which strengthens the analytical framework for sensitive biomonitoring. This can be attributed, at least in part, to the fractionation-based sample preparation, which yields cleaner samples by reducing potential interferences. It is also important to note that, due to the lack of a true blank urine matrix, LODs could not be determined for compounds that exhibited signals in blanks, indicating their presence in the matrix. Also, the repeatability and trueness were below the set criteria (<30% CV and <35% Bias). Normalized intensities of selected reference standards were tracked in QCs across each batch and plotted in QC charts (Figures S1-1–4), showing low deviations and confirming satisfactory method stability, though variations were somewhat higher in the HILIC approach.

A comprehensive set of 90 reference standards, spanning a broad polarity range, was utilized to assess the overall method performance and validate the dual-column approach. The true positive detection rate (TPR) was calculated as proposed by Fisher et al.,^33^ yielding a score of 0.90, respectively, with 81 out of 90 spiked test standards successfully detected. This high TPR demonstrates the method’s efficacy in accurately detecting known test standards across different polarities. Specifically, 48 test standards were identified as RP-specific, 5 as HILIC-specific, and 28 were detected by both instrumental approaches. Nine reference standards were however not detected, likely due to low ionization efficiency, mobile phase incompatibility, weak fragmentation, or high polarity/poor retention (e.g., tris(2-ethylhexyl) trimellitate, bisphenol A, ibuprofen, glyphosate).

### Nontargeted and Suspect Screening Results of the Real Samples

**Figure 2:**
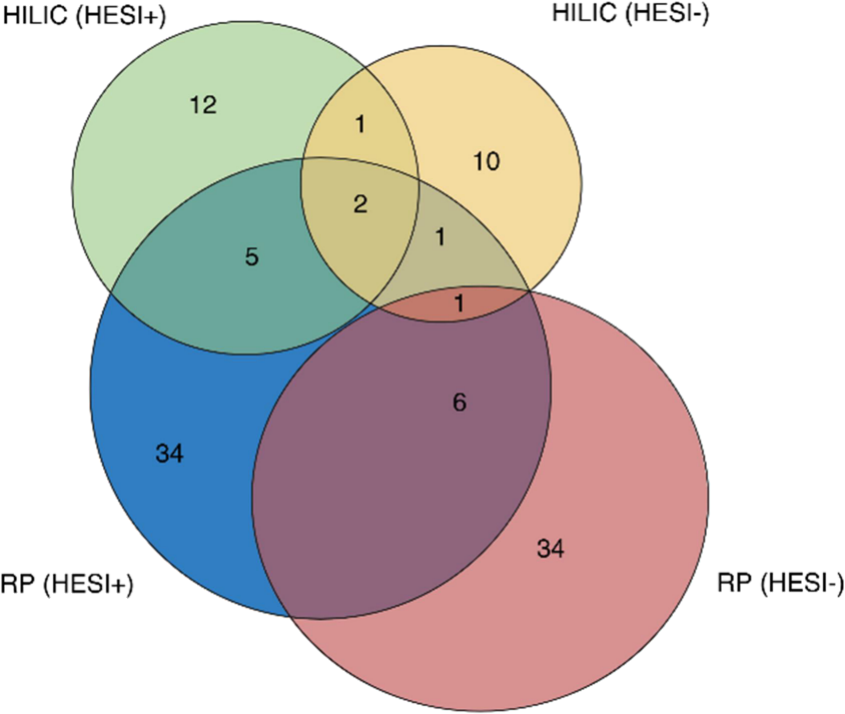
Venn diagram showing the number and overlap between BoEs detected with each instrumental method and annotated according to the criteria following Figure 1.

**Figure 3:**
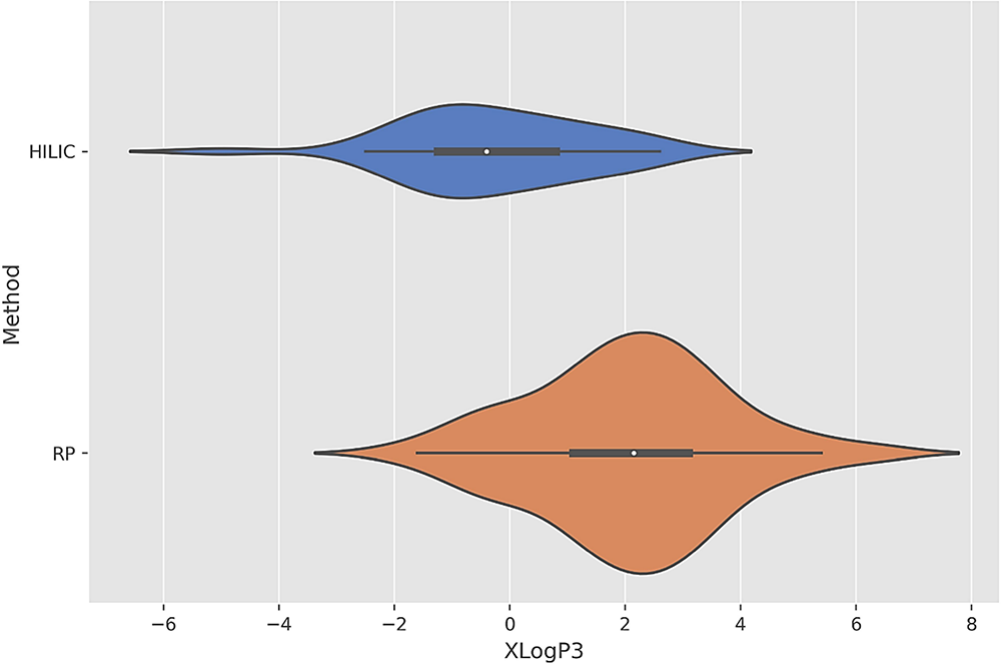
Polarity chemical space coverage: XLogP3 of BoEs, detected with HILIC and RP-based approaches.

Out of 106 annotated BoEs we classified 32 of them under level 1, 50 under level 2, and 24 under level 3, following the criteria given in Figure 1. The annotated BoEs are listed in Table S2-A4 and encompass a diverse range of compound classes, predominantly pharmaceuticals, plasticizers, and pesticides. The addition of the HILIC approach to the RP-based analysis resulted in the detection of 23 additional BoEs, representing a 28% increase in coverage. Additionally, 9 BoEs were detected by both approaches, indicating an overlap. The Venn diagram (Figure 2) illustrates the advantages of the dual-column strategy, highlighting unique and overlapping annotations across individual acquisition modes in HILIC and RP-based instrumental approaches. This demonstrates the broader chemical space achieved by combining both approaches. Moreover, the expansion of chemical space coverage is depicted in Figure 3, which plots the XLogP3 values against the number of BoEs detected using individual HILIC and RP-based approaches. The results strongly support the presented strategy, showing significantly wider coverage across different polarities compared to the traditional RP-based approach alone. This enhanced coverage is essential for comprehensive urinary profiling, particularly when aiming to detect low-abundance exogenous biomarkers that may have biological relevance to disease states such as OC.

Notably, the detection of 10 residual glucuronides between the detected BoEs (Table S2-A4) likely results from incomplete hydrolysis, as deconjugation efficiency depends on enzyme specificity, substrate complexity, and reaction conditions (pH, temperature, incubation time).^34^

**Figure 4:**
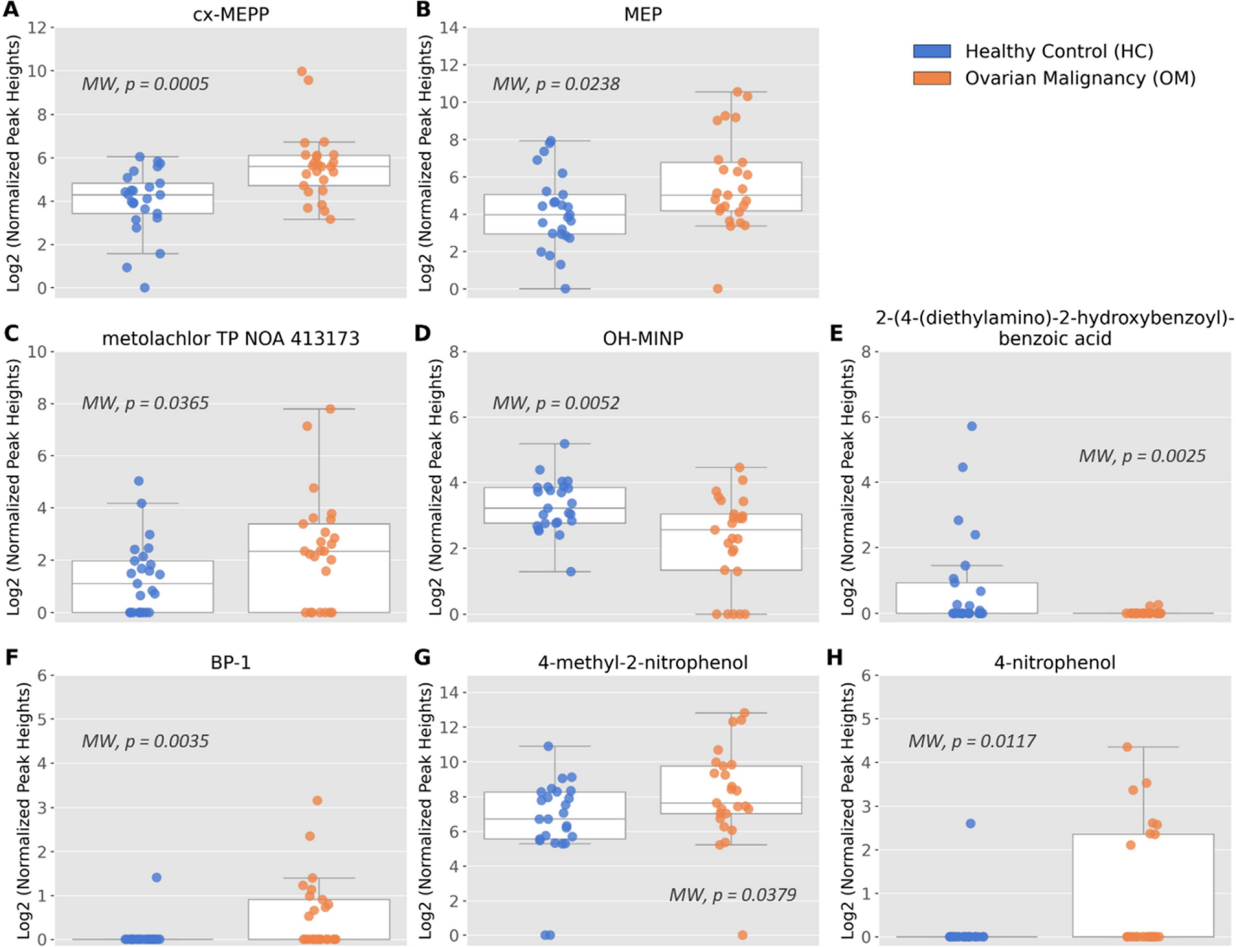
Box-Whisker plots (A–H) of Log2 normalized intensities for BoEs identified as having statistically significant differences with the Mann–Whitney test (*p* < 0.05) between OM (*n* = 25, orange) and HC (*n* = 25, blue).

A large share of the annotated biomarkers reflects lifestyle choices, such as consumption of caffeine and tobacco products, or the use of pharmaceuticals to treat or prevent symptoms or diseases. In contrast, industrial chemicals, pesticides, and other compounds primarily indicate involuntary exposure.^35^ Using the Mann–Whitney test (*p* < 0.05) we compared the differences in exposure to these chemicals and found significant differences in the normalized intensities of eight BoEs between the HC and OM individuals. The results are detailed in Table S2-A5 and are visualized in Figure 4. The results reveal significantly higher normalized intensities of mono(2-ethyl-5-carboxypentyl) phthalate (cx-MEPP), monoethyl phthalate (MEP), metolachlor TP NOA 413173, BP-1, 4-methyl-2-nitrophenol, and 4-nitrophenol (4-NP) in the OM group. Notably, these BoEs belong to phthalates, pesticides, and UV filters, many of which are known or suspected EDCs, potentially linking them to hormone cancers, such as OC.^36^ On the contrary, mono-(4-methyl-7-hydroxyoctyl) phthalate (OH-MINP), and 2-(4-(diethylamino)-2-hydroxybenzoyl)benzoic acid were higher in HC.

**Figure 5:**
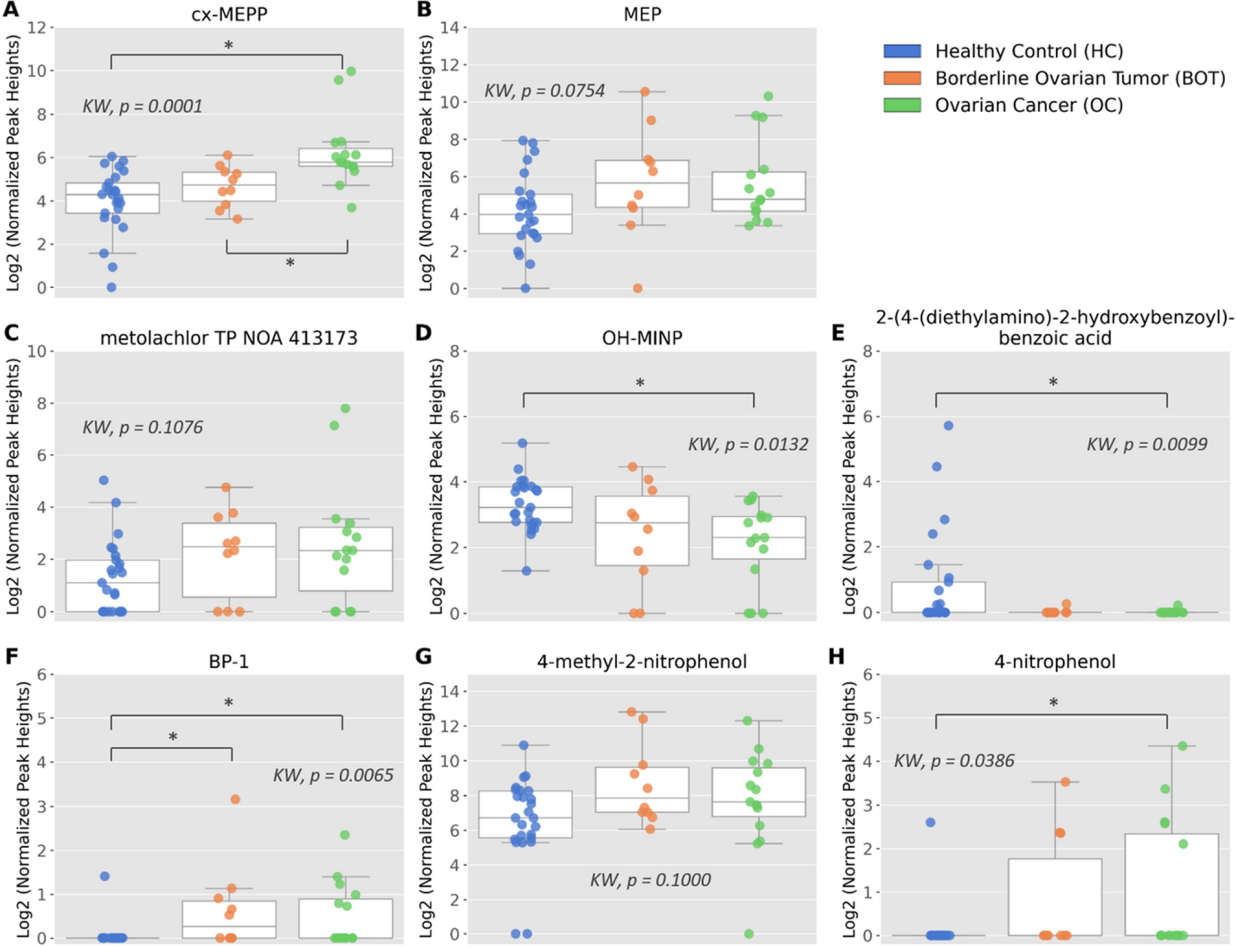
Box-and-whisker plots (A–H) of Log2 normalized intensities for the eight discriminatory BoEs in cancer subtypes (BOT, *n* = 10, orange; OC, *n* = 15, green) and HC (*n* = 25, blue). Statistically significant differences were tested using Kruskal–Wallis (KW) among three groups and the Mann–Whitney test between each two groups. Statistically significant differences (MW, *p* < 0.017) are indicated with an asterisk (*).

By further statistical analysis of the discriminatory BoEs, we aimed to find any potential differences related to cancer malignancy by comparing HC, BOT, and OC (*p* < 0.017). The results are visualized in Figure 5 with the corresponding data presented in Table S2-A6. Briefly, in women with OC, the normalized intensities of cx-MEPP, BP-1, and 4-NP in the urine were statistically significantly higher than in HC, indicating a higher exposure. Additionally, the normalized intensities of cx-MEPP were significantly elevated in OC compared to the BOT group. Also, in women with BOT, the normalized intensities of BP-1 in urine were statistically significantly higher than in HC. On the other hand, the normalized intensities of OH-MINP and 2-(4-(diethylamino)-2-hydroxybenzoyl)benzoic acid were statistically significantly lower in the urine of OC than in HC (Figure 5).

Below, we discuss the substances detected with statistically significant differences between women with OM and HC, along with their implications.

#### Phthalates and Their Metabolites

Phthalates (PHs) are extensively used in plastics, cosmetics, and pharmaceuticals,^37,38^ leading to pervasive food and environmental contamination. PHs and their metabolites have been detected in various human tissues and biological fluids.^39^ They are metabolized and excreted mainly through urine,^40,41^ with exposure linked to reproductive toxicity, endocrine disruption, and ovarian dysfunction.^42,43^ Diethylhexyl phthalate (DEHP) and its metabolite mono ethylhexyl phthalate (MEHP) are particularly well studied, with evidence of impaired ovarian function and increased metastasis risk in OC models.^5,44,45^ DEHP, dibutyl phthalate (DBP), di-isobutyl phthalate (DIBP), and benzyl butyl phthalate (BBP) are classified as toxic to reproduction, largely based on animal evidence (1B).^46^ In the present study, we observed that normalized intensities of cx-MEPP, a secondary metabolite of DEHP, were significantly elevated in OM patients as compared to HC (*p* < 0.05), suggesting higher exposure and potentially differing lifestyle habits. Additionally, cx-MEPP intensities were particularly elevated in the OC group, exhibiting a trend of OC > BOT > HC. This pattern indicates a possible association between DEHP exposure and OC development. However, further research is needed to confirm causality, taking into account also genetic susceptibility to phthalate exposure as a contributing factor.^47^ Likewise, the normalized intensities of MEP, the primary metabolite of diethyl phthalate (DEP), were elevated in OM patients, though its toxicity and OC relevance remain unclear.^48^ Conversely, OH-MINP, a secondary metabolite of diisononyl phthalate (DINP), was significantly lower in OM and OC patients compared to HC. While DINP exposure has been linked to ovarian dysfunction,^49^ direct evidence linking DINP exposure to OC is lacking.

Our results highlight significant differences in the normalized intensities of PHs metabolites, which serve as reliable indicators of PHs exposure. While these metabolites primarily reflect short-term exposure due to the rapid metabolism and excretion of PHs, their detection may also suggest chronic exposure. This could be attributed to lifestyle factors, indicating prolonged or even lifelong exposure to PHs.

#### Phytosanitary

Metolachlor TP NOA 413173, a metabolite of the chloroacetanilide herbicide metolachlor, and specifically its S stereoisomer (S-metolachlor) is currently classified as a suspected carcinogen by the European Chemicals Agency.^50^ Studies have linked metolachlor exposure to an increased risk of lymphoid malignancies^51^ and endocrine disruption.^52,53^ In our study, we observed substantially elevated normalized intensities of metolachlor TP NOA 413173 in the overall OM population, suggesting a possible link between metolachlor exposure and OC.

Similarly, 4-NP, a common intermediate in the synthesis of various chemicals and metabolite of now-banned organophosphate insecticides, showed elevated normalized intensities in the OM group, predominantly in OC patients. Though 4-NP exhibits estrogenic and antiandrogenic properties, its relevance to OC remains unclear.^54,55^

#### UV Filters

A large group of benzophenone derivatives are commonly found in sunscreens and other cosmetic products, leading to ubiquitous human exposure.^56^ They have been associated with endocrine disruption, particularly affecting thyroid and sex hormones, and potential carcinogenicity.^10,57^ Both BOT and OC subgroups exhibited elevated normalized intensities of BP-1 as compared to HC, which may reflect increased exposure, altered metabolism, or accumulation in tumor tissues.

Further, 2-(4-(diethylamino)-2-hydroxybenzoyl)benzoic acid is likely the metabolite of a UV filter, 2-(4-(diethylamino)-2-hydroxybenzoyl)benzoate (DHHB), another discriminatory BoE found in OM and HC samples.^58^ Although no studies have directly associated DHHB with endocrine-disrupting properties, similar compounds within the same chemical class exhibit such effects, suggesting the need for further investigation. Our results show significantly higher normalized intensities of 2-(4-(diethylamino)-2-hydroxybenzoyl)benzoic acid in the HC samples as compared to the OM group. Additionally, normalized intensities were significantly lower in the OC subgroup compared to the HC group. This can be explained by the lower exposure to the parent compound, but also by possible metabolic alterations associated with OC, leading to possible altered metabolism and/or excretion.

#### Other BoEs

4-methyl-2-nitrophenol is an industrial intermediate with limited toxicity data, which was elevated in OM patients. Its structural similarity to known hazardous nitrophenols suggests a need for further investigation.

### Interpreting Biomarker Variations

The observed differences in normalized BoEs intensities between OM and HC suggest complex interactions between environmental exposures and cancer onset. Elevated normalized intensities of certain BoEs in cancer patients may not necessarily indicate a causal relationship but could also reflect lifestyle changes, altered metabolism, or tumor-related processes, which were not specifically assessed in this study. This consideration is crucial, as severe malignancies such as OC are often accompanied by significant lifestyle changes, leading to altered exposure profiles. A similar dynamic applies to comparisons between patients with BOT and OC. A notable limitation in the study design is the composition of the HC cohort, which included pregnant women. Pregnancy involves substantial physiological changes, including altered metabolism and kidney function, which differ from the general population. However, unlike women with OM, these individuals had healthy ovaries with normal function, enabling successful pregnancy. The relatively small sample size in each group presents another limitation, though patient recruitment was carefully controlled to ensure group validity. Overall, while findings suggest possible links between specific exposures and OC, they do not establish causality.

This study successfully utilized a fractionation-based sample preparation and combined HILIC and RP-UHPLC-HRMS to profile the urinary exposome of women with OM, uncovering significant differences in specific EDCs compared to HC.

Elevated normalized intensities of cx-MEPP, MEP, metolachlor TP NOA 413173, and BP-1 reinforce concerns about the potential role of these ubiquitous BoEs in hormone-related cancers. Notably, cx-MEPP normalized intensities correlated with malignancy severity, suggesting a possible link between DEHP exposure and cancer development. Additionally, the use of open-source software for data processing enhances accessibility and reproducibility, promoting their broader adoption.

With minor adaptations, this approach could be applied to various biological, environmental, and food matrices, further supporting PARC’s mission to establish robust tools for chemical monitoring and early warning systems for compounds of emerging concern. While these findings offer valuable insights, further research with larger, more diverse cohorts and longitudinal analyses is needed to confirm these findings, refining our understanding of the relationship between environmental exposures and OC development.

## Supporting Information Available

Materials and chemicals, data processing parameters, quality control charts, standard class and XLogP3 distribution chart, SPE optimization results charts, and specific gravity adjustment (PDF)
Table of reference standards, table of system control reference standards and validation results, table of identified BoEs, and statistical analysis results tables (XLSX)
Mass spectral library: positive.mgf (PDF)
negative.mgf (PDF)

## Abbreviations

ACN: acetonitrile
AU: arbitrary unit
BOT: borderline ovarian tumors
CV: coefficient of variation
DMSO: dimethyl sulfoxide
EDCs: endocrine-disrupting compounds
EtOAc: ethyl acetate
HC: healthy controls
HCD: higher-energy collisional dissociation
HESI: heated electrospray ionization
HILIC: hydrophilic interaction liquid chromatography
HRMS: high-resolution mass spectrometry
IS: internal standard
LOD: lower limit of detection
MeOH: methanol
MS/MS: tandem mass spectrometry
NTS: nontargeted screening
OC: ovarian cancer
OM: ovarian malignancies
QC: quality control
RP: reversed-phase
SPE: solid-phase extraction
SS: suspect screening
TPR: true positive detection rate
UHPLC: ultrahigh-performance liquid chromatography
