# Supplementary material for: Nontargeted Urinary Profiling Strategy for Endocrine-Disrupting Chemicals in Women with Ovarian Malignancies
Source: Environ Sci Technol. 2025 Apr 22;59(17):8380–90. doi: 10.1021/acs.est.4c13290 (PMC12060279; doi:10.1021/acs.est.4c13290)
Supplement: Supplementary file 1 — es4c13290_si_001.pdf [file es4c13290_si_001.pdf]

Nontargeted Urinary Profiling Strategy for Endocrine-Disrupting Chemicals in Women with Ovarian Malignancies

Helena Plešnik[1,2‡], Žan Rekar[1,2‡], Stefanela Stevanović[3], Irma Virant-Klun[4,5], Senka Imamović Kumalić[5,6], Mateja Sladič[5,6], Darja Mazej[1], Janja Snoj Tratnik[1], Milena Horvat[1,2], Tina Kosjek[1,2*]

[1] *Department of Environmental Sciences, Jožef Stefan Institute, 1000 Ljubljana, Slovenia*

[2] *Jožef Stefan International Postgraduate School, 1000 Ljubljana, Slovenia*

[3] *Faculty of Computer and Information Science, University of Ljubljana, 1000 Ljubljana, Slovenia*

[4] *Clinical Research Centre, University Medical Centre Ljubljana, 1000 Ljubljana, Slovenia*

[5] *Faculty of Medicine, University of Ljubljana, 1000 Ljubljana, Slovenia*

[6] *Division of Obstetrics and Gynecology, University Medical Centre Ljubljana, 1000 Ljubljana, Slovenia*

‡ These two authors contributed equally to this work

* Corresponding author contact: tina.kosjek@ijs.si

Summary: 11 pages, 4 texts, 8 figures, 6 tables (in Excel file).

Contents

1. Materials and Chemicals 2
2. Data preprocessing 2
   2.1. HILIC-based separation method 2
   2.2. RP-based separation method 2
3. Quality assurance and quality control 3
4. Sample preparation development 8
5. Specific gravity adjustment 11

1. Materials and Chemicals

Sodium acetate buffer solution (pH 5.2, 3M), β-glucuronidase/arylsulfatase solution (Helix Pomatia, type H-2, aqueous solution, ≥ 85,000 units/mL), and dimethyl sulfoxide were purchased from Sigma Aldrich (St Louis, MO, USA). LC-MS-grade acetonitrile (ACN), LC-MS-grade methanol (MeOH), LC-MS-grade water, and BAKER ULTRA RESI-ANALYZED ethyl acetate were purchased from J. T. Baker (Phillipsburg, NJ, USA). Oasis HLB 96-well plates (60 mg) and collection plates were obtained from Waters Corporation (Milford, MA, USA). RC Membrane Syringe filters (0.2 μm) were purchased from Phenomenex (Torrance, CA, USA).

The software used included Python programming language (v3.11.5), Xcalibur (Thermo Fisher Scientific, Waltham, MA, USA), and Biorender (BioRender, Toronto, Canada). Data analysis was performed in the Jupyter Notebook environment, with visualizations created using the Matplotlib and Seaborn libraries.

2. Data preprocessing

2.1. HILIC-based separation method

The following steps were applied in data processing with mzmine 4.3.0 for RP-based separation method: (i) Mass detection at the noise level of 50000 for MS1 and 25000 for MS2, (ii) Chromatogram builder at the minimum 5 consecutive scans, minimum intensity for consecutive scans of 50000, minimum absolute height of 50000, and the m/z tolerance of 0.0020 m/z or 10.0 ppm, (iii) Retention time Smoothing using Savitzky Golay algorithm, (iv) Local minimum feature resolver with the 85% chromatographic threshold, minimum RT search range of 0.15 min, minimum absolute height of 50000, the minimum ratio of peak top/edge of 2.0, peak duration range of 0.00–4.51 and minimum 4 scans of a feature, (v) 13C isotope filter with the m/z tolerance of 3.0 ppm, RT tolerance of 0.20 min, maximum charge of 1 and with the most intense isotope set as the representative isotope, (vi) Join aligner with the m/z tolerance of 0.0015 m/z or 5.0 ppm and the weight for of 2, the RT tolerance of 0.5 min and the weight of 1 and with no requirement of charge state or ID and no comparison of isotope pattern and spectra similarity, (vii) Peak finder using 1% intensity tolerance, m/z tolerance of 0.0010 m/z or 5.0 ppm, RT tolerance of 0.5 min, and minimum 4 scans, (viii) Duplicate peak filter with new average filter mode, m/z tolerance of 0.0008 m/z or 1.5 ppm, and RT tolerance of 0.10 min.

2.2. RP-based separation method

The following steps were applied in data processing with mzmine 4.3.0 for RP-based separation method: (i) Mass detection at the noise level of 10000 for MS1 and 1000 for MS2, (ii) Chromatogram builder at the minimum 4 consecutive scans, minimum intensity for consecutive scans of 10000, minimum absolute height of 30000, and the m/z tolerance of 0.0010 m/z or 5.0 ppm, (iii) Retention time Smoothing using Savitzky Golay algorithm, (iv) Local minimum feature

resolver with the 92% chromatographic threshold, minimum RT search range of 0.05 min, minimum absolute height of 10000, the minimum ratio of peak top/edge of 2.70, peak duration range of 0.02–1.00 and minimum 4 scans on a feature, (v) 13C isotope filter with the m/z tolerance of 3.0 ppm, RT tolerance of 0.12 min, maximum charge of 1 and with the most intense isotope set as the representative isotope, (vi) Join aligner with the m/z tolerance of 0.0010 m/z or 5.0 ppm and the weight for of 2, the RT tolerance of 0.15 min and the weight of 1 and with no requirement of charge state or ID and no comparison of isotope pattern and spectra similarity, (vii) Peak finder using 1% intensity tolerance, m/z tolerance of 0.0010 m/z of 5.0 ppm, RT tolerance of 0.12 min, and minimum 4 scans, (viii) Duplicate peak filter with new average filter mode, m/z tolerance of 0.0008 m/z or 1.5 ppm, and RT tolerance of 0.04 min.

3. Quality assurance and quality control

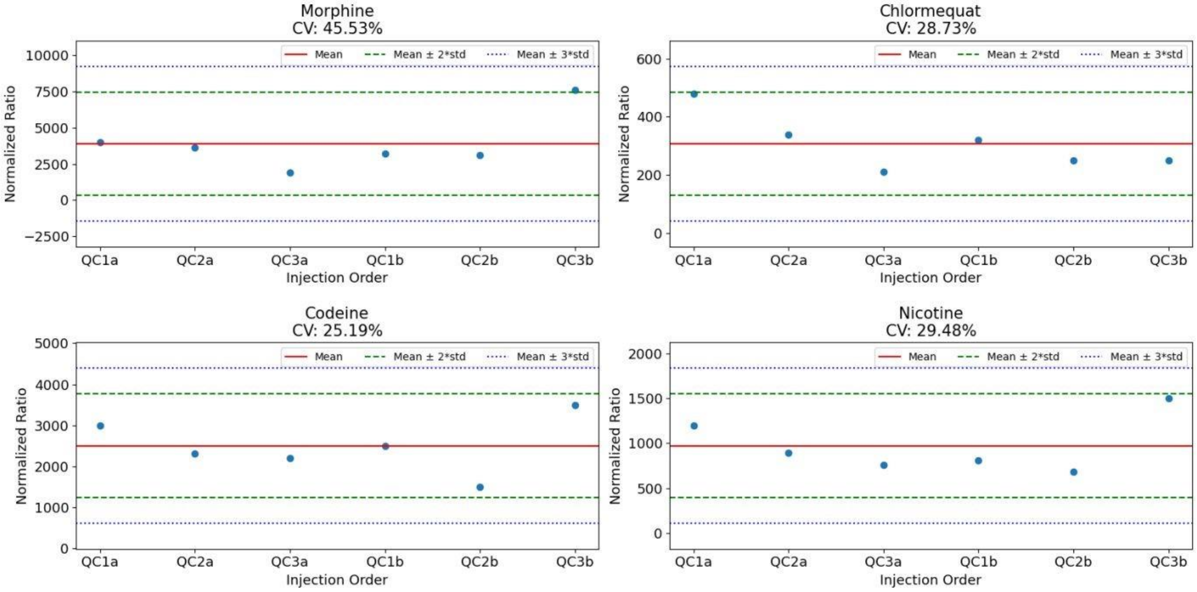

Figure S1-1: Deviations of the selected compounds' normalized intensities in QCs over the course of the analytical run, HILIC-based separation analyses (ESI+). The upper and lower limits were calculated based on standard deviation of all 6 measurements.

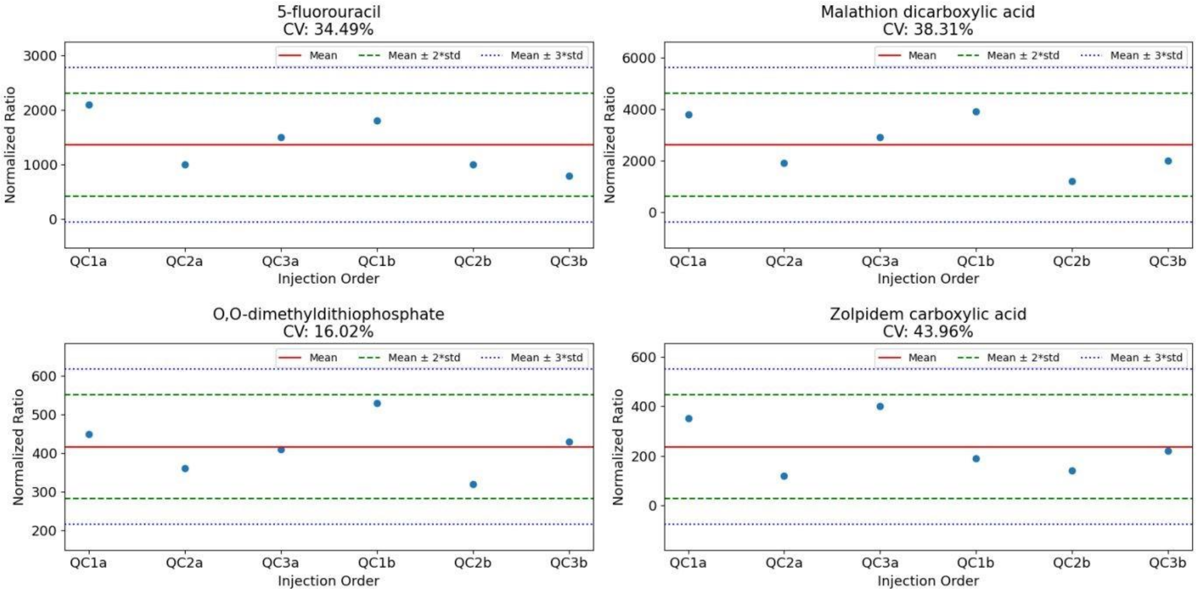

Figure S1-2: Deviations of the selected compounds' normalized intensities in QCs over the course of the analytical run, HILICbased separation analyses (ESI-). The upper and lower limits were calculated based on standard deviation of all 6 measurements.

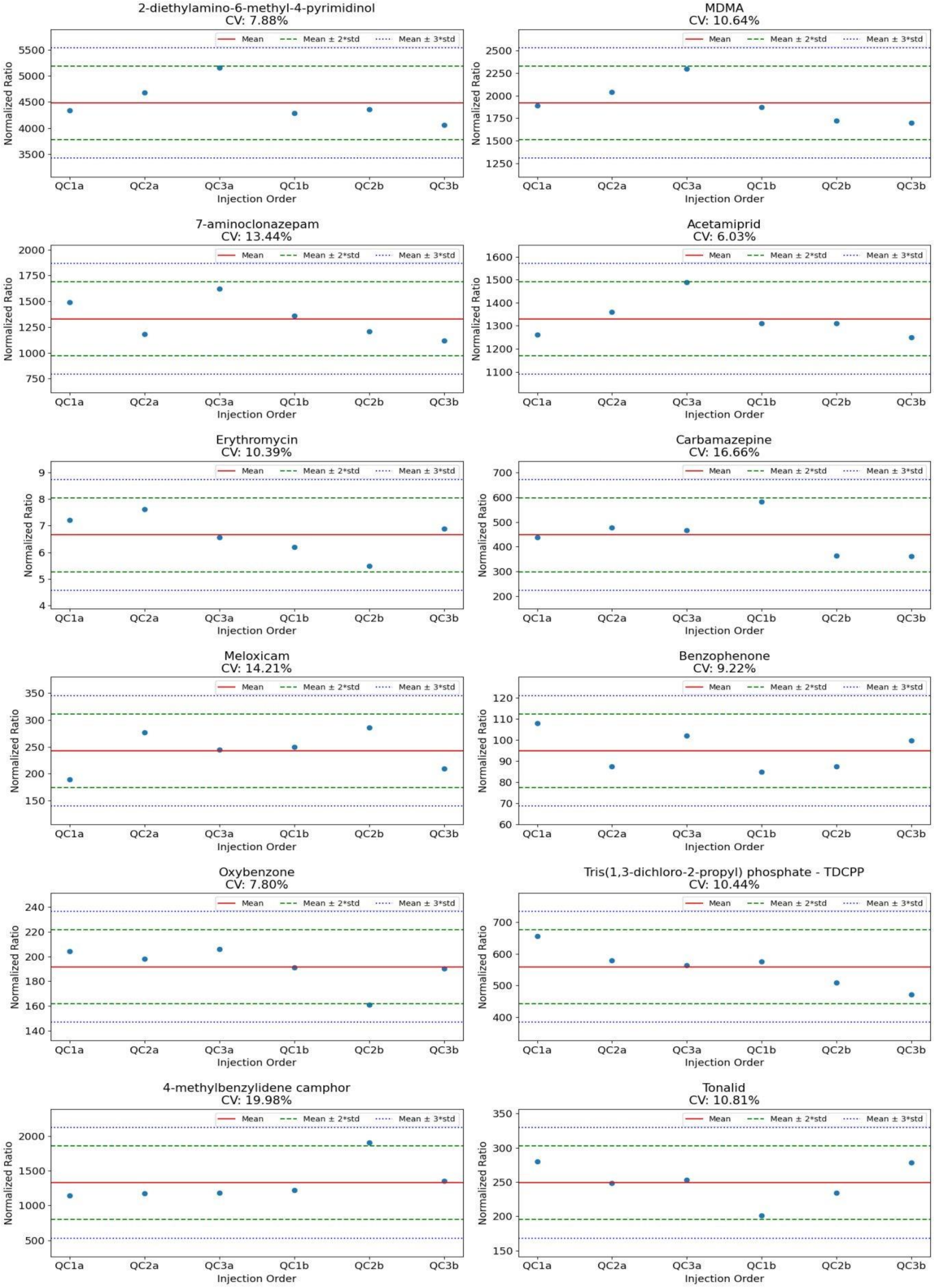
2-diethylamino-6-methyl-4-pyrimidinol
CV: 7.88%
MDMA
CV: 10.64%
7-aminoclonazepam
CV: 13.44%
Acetamiprid
CV: 6.03%
Erythromycin
CV: 10.39%
Carbamazepine
CV: 16.66%
Meloxicam
CV: 14.21%
Benzophenone
CV: 9.22%
Oxybenzone
CV: 7.80%
Tris(1,3-dichloro-2-propyl) phosphate - TDCPP
CV: 10.44%
4-methylbenzylidene camphor
CV: 19.98%
Tonalid
CV: 10.81%
Mean
Mean ± 2*std
Mean ± 3*std
Normalized Ratio
Injection Order
QC1a
QC2a
QC3a
QC1b
QC2b
QC3b

Figure S1-3: Deviations of the selected compounds' normalized intensities in QCs over the course of the analytical run, RPbased separation analyses (ESI+). The upper and lower limits were calculated based on standard deviation of all 6 measurements.

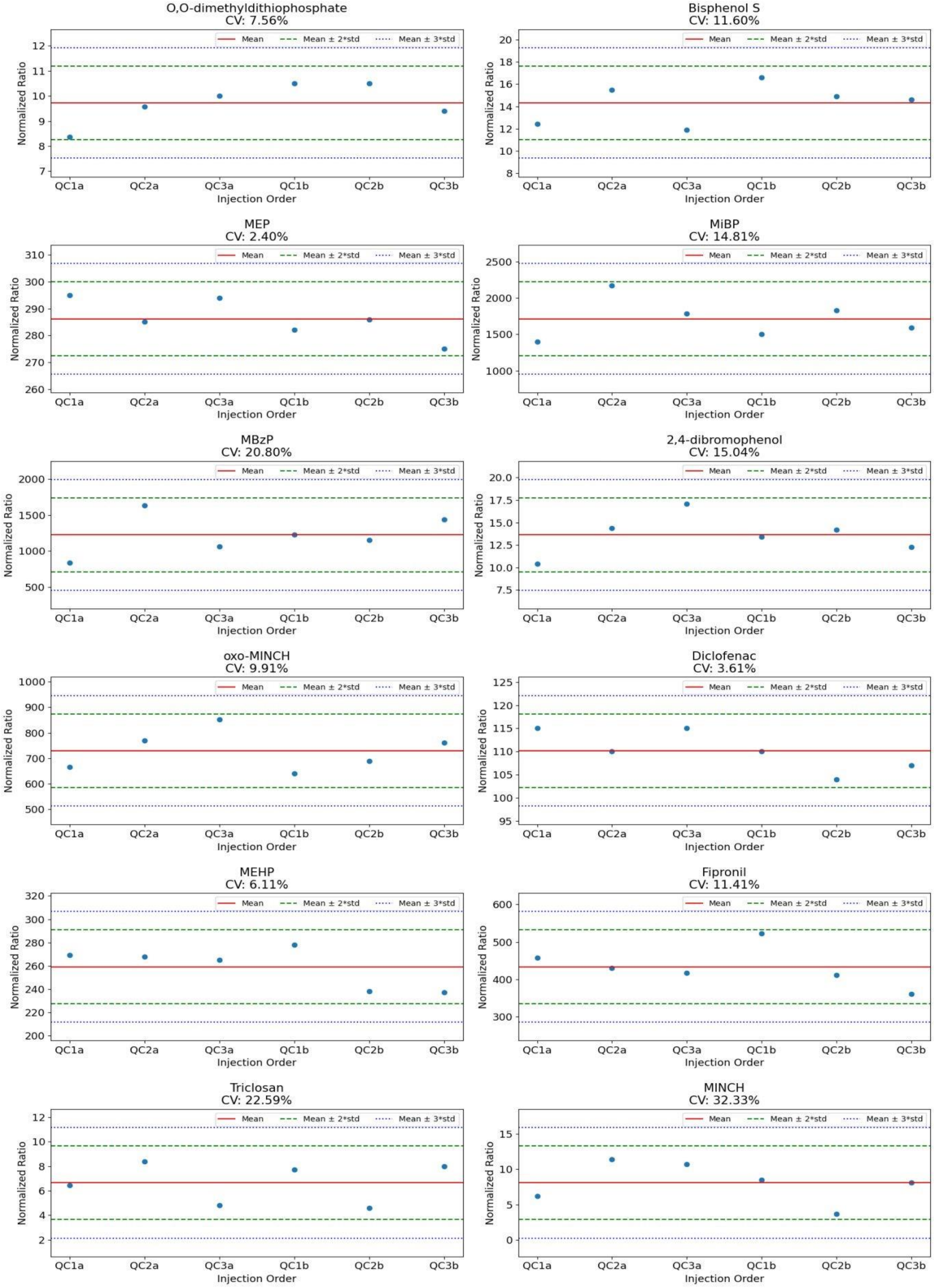
O,O-dimethyldithiophosphate
CV: 7.56%
Bisphenol S
CV: 11.60%
MEP
CV: 2.40%
MiBP
CV: 14.81%
MBzP
CV: 20.80%
2,4-dibromophenol
CV: 15.04%
oxo-MINCH
CV: 9.91%
Diclofenac
CV: 3.61%
MEHP
CV: 6.11%
Fipronil
CV: 11.41%
Triclosan
CV: 22.59%
MINCH
CV: 32.33%
Mean
Mean ± 2*std
Mean ± 3*std
Normalized Ratio
Injection Order
QC1a
QC2a
QC3a
QC1b
QC2b
QC3b

Figure S1-4: Deviations of the selected compounds' normalized intensities in QCs over the course of the analytical run, RPbased separation analyses (ESI-). The upper and lower limits were calculated based on standard deviation of all 6 measurements.

4. Sample preparation development

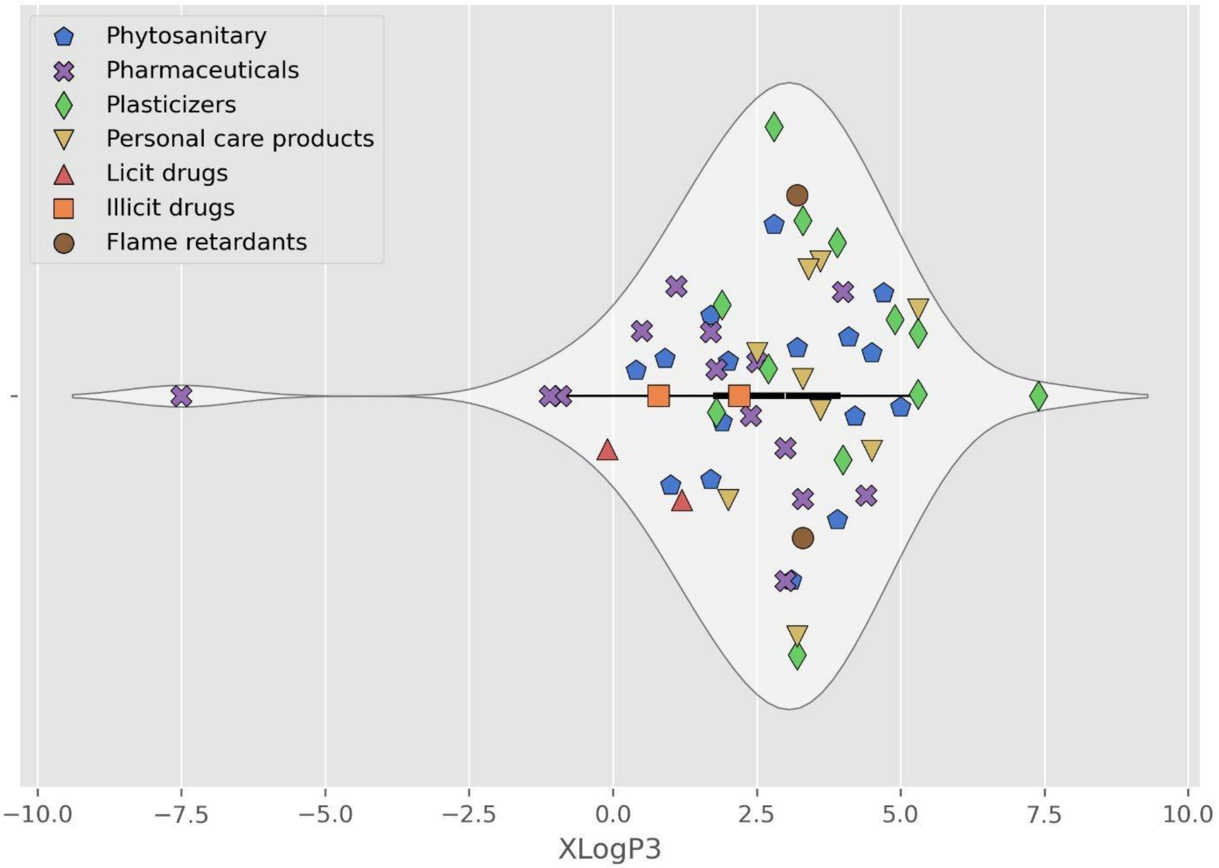

Figure S1-5: Sample preparation standard mix: Compound class and XLogP3 distribution of selected reference standards.

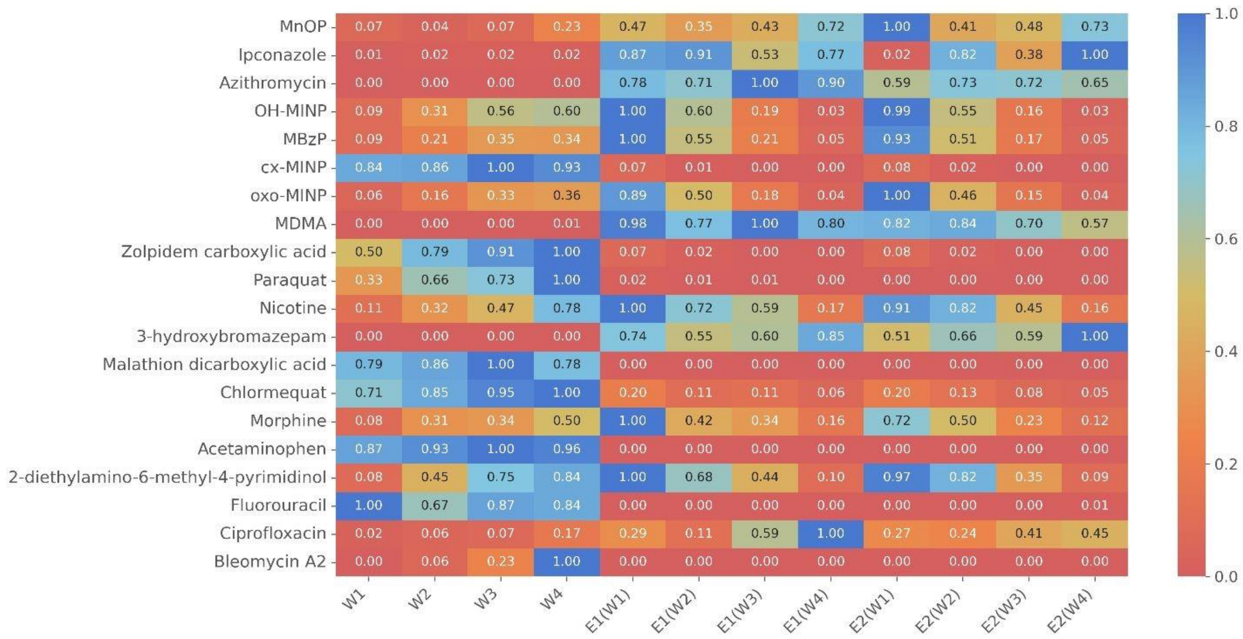

Figure S1-6: SPE optimization: Heatmap of all detected reference standards with normalized intensities scaled between 0 and 1 across different eluents (wash and elution fractions of different solvent compositions) analyzed by HILIC-based separation method; **W1**: Wash 1 (20% MeOH in H_2O), **W2**: Wash 2 (30% MeOH in H_2O), **W3**: Wash 3 (40% MeOH in H_2O), **W4**: Wash 4 (50% MeOH in H_2O), **E1**: Elution 1 (10% EtOAc in ACN), **E2**: Elution 2 (30% EtOAc in ACN); **E1(W1)**: Elution 1 after Wash 1, **E1(W2)**: Elution 1 after Wash 2, **E1(W3)**: Elution 1 after Wash 3, **E1(W4)**: Elution 1 after Wash 4, **E2(W1)**: Elution 2 after Wash 1, **E2(W2)**: Elution 2 after Wash 2, **E2(W3)**: Elution 2 after Wash 3, **E2(W4)**: Elution 2 after Wash 4; Reference standards are sorted from top to bottom in order of decreasing XLogP3 value.

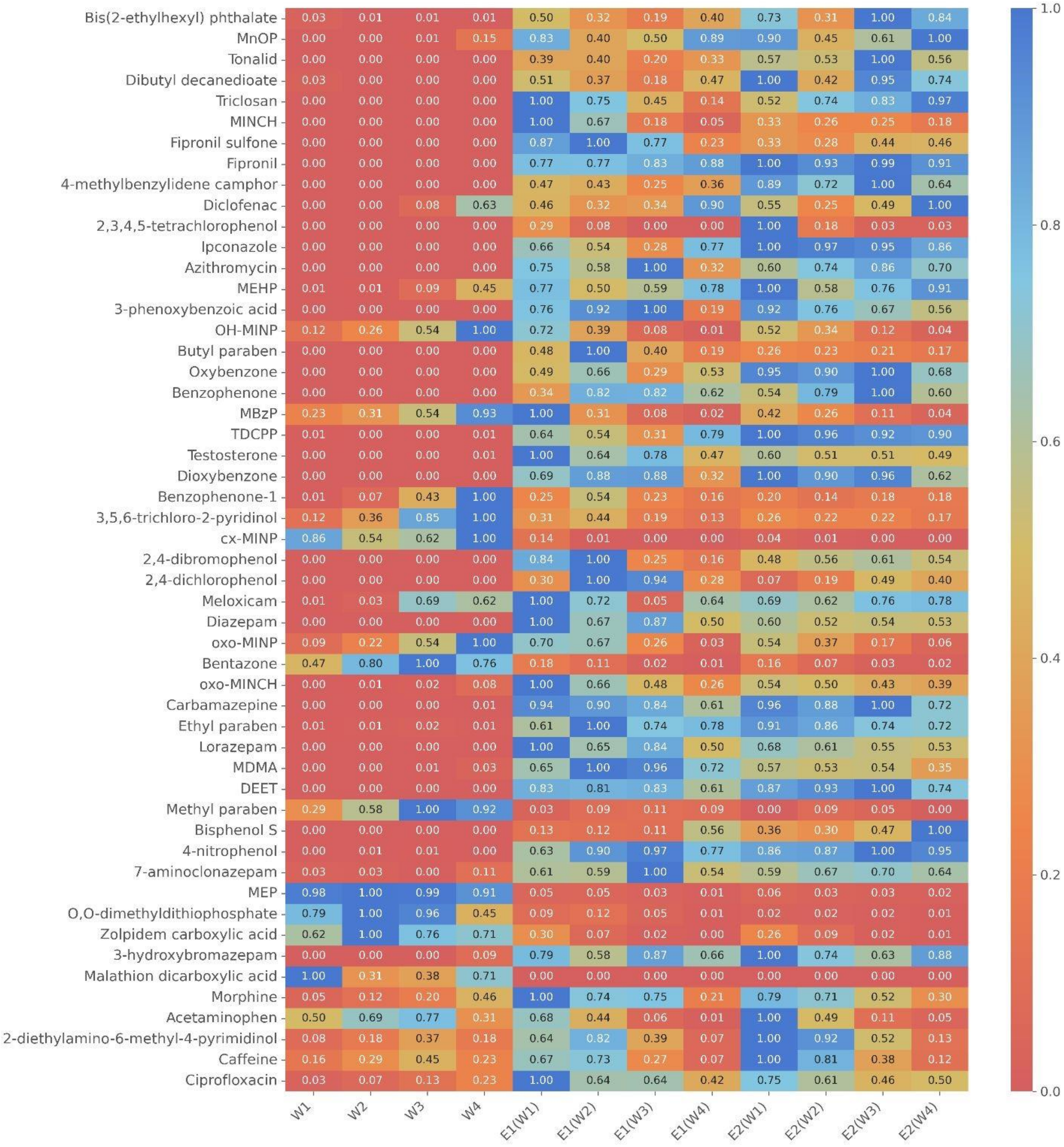

Figure S1-7: SPE optimization: : Heatmap of all detected reference standards with normalized intensities scaled between 0 and 1 across different eluents (wash and elution fractions of different solvent compositions) analyzed by RP-based separation method; **W1**: Wash 1 (20% MeOH in H_2O), **W2**: Wash 2 (30% MeOH in H_2O), **W3**: Wash 3 (40% MeOH in H_2O), **W4**: Wash 4 (50% MeOH in H_2O), **E1**: Elution 1 (10% EtOAc in ACN), **E2**: Elution 2 (30% EtOAc in ACN); **E1(W1)**: Elution 1 after Wash 1, **E1(W2)**: Elution 1 after Wash 2, **E1(W3)**: Elution 1 after Wash 3, **E1(W4)**: Elution 1 after Wash 4, **E2(W1)**: Elution 2 after Wash 1, **E2(W2)**: Elution 2 after Wash 2, **E2(W3)**: Elution 2 after Wash 3, **E2(W4)**: Elution 2 after Wash 4; Reference standards are sorted from top to bottom in order of decreasing XLogP3 value.

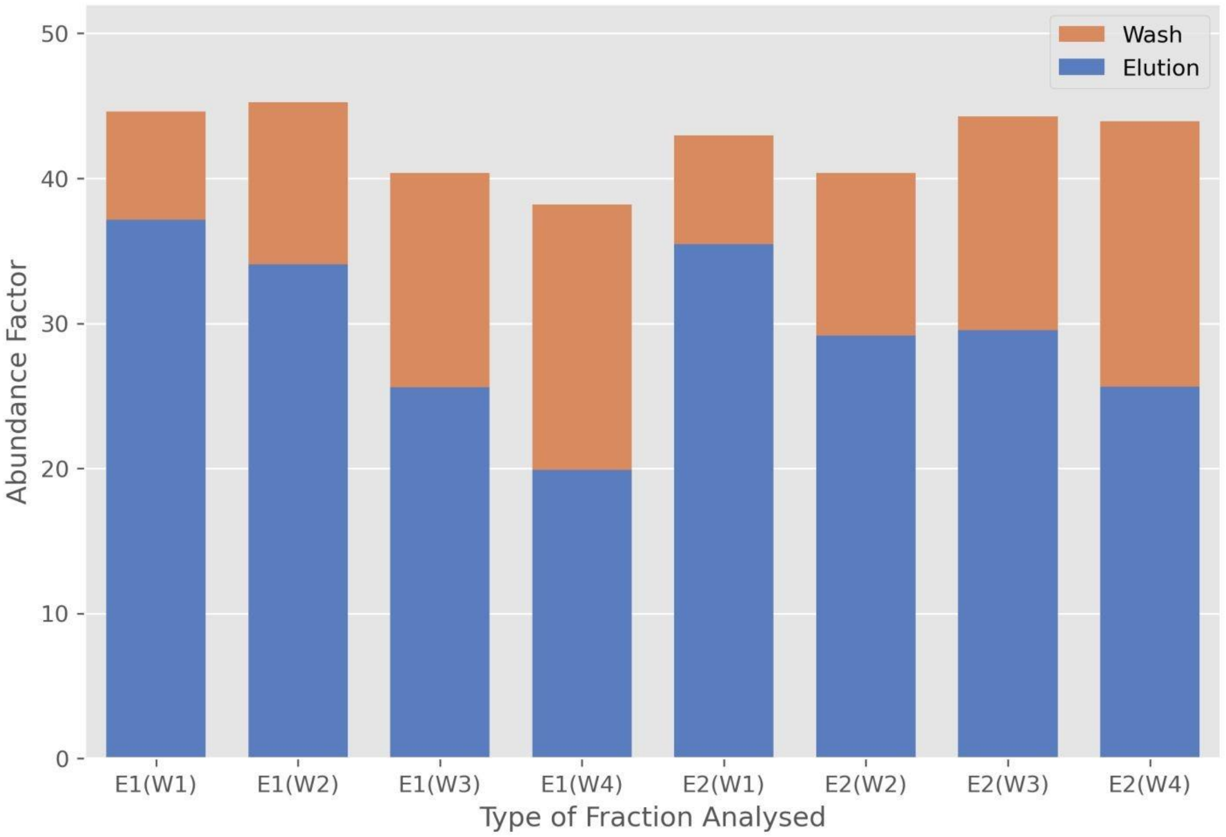

Figure S1-8: Bar chart representing abundance factor for each tested wash/elution combination combining results of elution fraction obtained with RP-based separation method and results of wash fraction obtained with HILIC-based separation method; **W1**: Wash 1 (20% MeOH in H_2O), **W2**: Wash 2 (30% MeOH in H_2O), **W3**: Wash 3 (40% MeOH in H_2O), **W4**: Wash 4 (50% MeOH in H_2O), **E1**: Elution 1 (10% EtOAc in ACN), **E2**: Elution 2 (30% EtOAc in ACN); **E1(W1)**: Elution 1 after Wash 1, **E1(W2)**: Elution 1 after Wash 2, **E1(W3)**: Elution 1 after Wash 3, **E1(W4)**: Elution 1 after Wash 4, **E2(W1)**: Elution 2 after Wash 1, **E2(W2)**: Elution 2 after Wash 2, **E2(W3)**: Elution 2 after Wash 3, **E2(W4)**: Elution 2 after Wash 4.

5. Specific gravity adjustment

To account for urine sample dilution the normalized intensities were corrected using specific gravity (SG) adjustment. The SG was determined using a PAL-10S refractometer (Atago®, Japan), with a measurement range of 1.000–1.060. SG-corrected normalized intensities were calculated by the formula:

$C_{adjusted}$ (µg/L) = $C_{measured} \times (SG_s - 1) / (SG_i - 1)$

where $C_{adjusted}$ is the adjusted normalized intensity, $C_{measured}$ is measured normalized intensity, SG_s is standard specific gravity, which was calculated as average specific gravity (SG_s = 1.012), and SG_i is measured specific gravity of an individual sample.
