# Supplementary material for: Nontargeted Urinary Profiling Strategy for Endocrine-Disrupting Chemicals in Women with Ovarian Malignancies
Source: Environ Sci Technol. 2025 Apr 22;59(17):8380–90. doi: 10.1021/acs.est.4c13290 (PMC12060279; doi:10.1021/acs.est.4c13290)
Supplement: Supplementary file 3 — es4c13290_si_003.pdf [file es4c13290_si_003.pdf]

```
BEGIN IONS
NAME=11-Nor-9-carboxy-thc
INCHIAUX=
MSLEVEL=MS2
INSTRUMENT_TYPE=Orbitrap
SOURCE_INSTRUMENT=
IONMODE=Positive
Collision energy=
FORMULA=C21H28O4
EXACTMASS=0.0
PEPMASS=345.2063
ADDUCT=M+H
65.038658 13135.4375
67.054321 11437.5322265625
69.069977 25753.419921875
77.03862 10530.24609375
79.054268 18620.708984375
91.054169 34817.796875
93.069763 12755.23046875
95.049042 23225.671875
95.085457 12653.4873046875
105.044617 11507.619140625
115.054153 15024.9072265625
119.085487 49500.6875
123.044029 32917.48828125
161.059723 10690.5048828125
161.096146 17398.1640625
165.091034 14141.7607421875
187.075394 32762.240234375
193.122314 86070.421875
202.077698 36028.33984375
207.137817 10465.55859375
217.122269 22973.70703125
229.12233 25291.125
231.137909 20580.1640625
243.137985 14866.818359375
257.117493 16122.6513671875
257.153717 19289.41015625
271.132935 12636.6015625
281.190155 10857.078125
299.200684 198545.140625
327.195709 185507.671875
345.206329 59350.06640625
END IONS


BEGIN IONS
NAME=2-Diethylamino-6-methyl-4-pyrimidinol
INCHIAUX=
MSLEVEL=MS2
INSTRUMENT_TYPE=Orbitrap
SOURCE_INSTRUMENT=
IONMODE=Positive
Collision energy=
```

FORMULA=C9H15N3O
EXACTMASS=0.0
PEPMASS=182.1288
ADDUCT=M+H
43.029095 132675.484375
45.532433 15293.9765625
53.156006 10102.650390625
54.03389 18168.4453125
59.049156 10551.2265625
67.017952 23777.521484375
67.029144 10641.5849609375
68.013168 39629.36328125
69.008392 108891.84375
71.060493 47948.515625
74.096458 28410.5
74.732407 10227.6884765625
84.044373 397440.96875
94.699768 10696.8994140625
99.091553 110697.3984375
109.039528 98906.8359375
110.885048 10170.68359375
112.075508 15249.1298828125
126.066101 80017.4453125
135.90387 11881.4052734375
137.070953 134533.0625
154.097534 990024.125
155.101135 12887.9794921875
180.11322 17211.947265625
182.128754 3259954.25
182.139282 24636.7578125
183.131958 39834.89453125
202.077698 211268.75
203.080826 12271.99609375
203.085953 11987.333984375
END IONS

BEGIN IONS
NAME=3-Hydroxybromazepam
INCHIAUX=
MSLEVEL=MS2
INSTRUMENT_TYPE=Orbitrap
SOURCE_INSTRUMENT=
IONMODE=Positive
Collision energy=
FORMULA=C14H10BrN3O2
EXACTMASS=0.0
PEPMASS=332.0031
ADDUCT=M+H
51.022957 9781.4775390625
78.033852 18822.69921875
79.041695 4412.779296875
93.057236 6784.9033203125
96.044266 82095.4375

```
105.044556 11323.6103515625
124.039207 26294.00390625
171.975662 3804.661865234375
179.06041 6939.79638671875
182.083832 11100.45703125
202.077698 29816.51171875
207.079086 6577.94580078125
208.063354 5298.2734375
224.081711 5815.57177734375
224.965729 7917.7099609375
258.986603 6775.2734375
261.002075 6873.74365234375
273.997498 8079.94677734375
276.01297 4139.10302734375
285.997406 4677.25146484375
286.981506 107646.859375
288.151093 3936.4326171875
303.000214 54471.8671875
313.992432 26404.361328125
314.97644 43726.7734375
332.00293 21263.1953125
332.140533 4032.26708984375
END IONS


BEGIN IONS
NAME=7-Aminoclonazepam
INCHIAUX=
MSLEVEL=MS2
INSTRUMENT_TYPE=Orbitrap
SOURCE_INSTRUMENT=
IONMODE=Positive
Collision energy=
FORMULA=C15H12ClN3O
EXACTMASS=0.0
PEPMASS=286.0744
ADDUCT=M+H
51.023003 51064.53125
65.038666 107688.4921875
67.054306 41199.75390625
75.023041 55539.65625
77.03862 146078.484375
78.033844 32757.6328125
80.049477 93803.109375
82.052536 146439.6875
92.049347 51590.87109375
93.057213 120557.28125
94.065048 181619.359375
95.049034 230880.6875
102.033745 31139.47265625
104.049393 86083.1484375
105.04464 180508.4375
107.060249 31314.755859375
115.054237 44172.0625
```

119.060196 37448.52734375
121.075989 981090.6875
130.039932 33002.59765625
139.05426 81881.2421875
140.049591 48881.4375
146.07135 61590.0625
147.05545 43737.87109375
149.070908 74099.34375
150.046509 30850.55078125
152.062195 73049.21875
154.064987 30601.51953125
166.065216 79136.2109375
167.073013 113747.2421875
169.064789 76394.984375
177.057297 59694.8359375
178.065186 57184.421875
194.083893 102307.6328125
195.091553 105498.203125
202.077698 489120.78125
203.08049 65991.6953125
203.085785 44833.76953125
205.075928 80846.5234375
209.094757 131132.25
222.102539 1068423.875
242.060715 34285.05859375
250.097488 1169644.375
258.079102 114547.140625
286.053955 37682.6796875
286.07431 5452320.5
286.095398 33222.703125
287.077942 41348.5859375
END IONS

BEGIN IONS
NAME=Acetaminophen
INCHIAUX=
MSLEVEL=MS2
INSTRUMENT_TYPE=Orbitrap
SOURCE_INSTRUMENT=
IONMODE=Positive
Collision energy=
FORMULA=C8H9NO2
EXACTMASS=0.0
PEPMASS=152.0707
ADDUCT=M+H
43.017864 101995.671875
65.038666 455011.125
67.041748 21849.568359375
80.049538 27236.291015625
82.065147 15696.1376953125
92.049446 56917.609375
93.033417 44028.875
93.057228 27597.435546875

```
109.052116 34404.27734375
110.054932 15479.8046875
110.059944 2363031.0
110.064934 14177.6357421875
111.043922 91532.8671875
111.063332 32591.498046875
134.060028 43716.72265625
152.062592 13978.4853515625
152.070663 2072131.0
152.076859 16049.3486328125
152.078674 11677.6220703125
153.06601 11414.1142578125
153.073883 33621.7578125
END IONS


BEGIN IONS
NAME=Acetamiprid
INCHIAUX=
MSLEVEL=MS2
INSTRUMENT_TYPE=Orbitrap
SOURCE_INSTRUMENT=
IONMODE=Positive
Collision energy=
FORMULA=C10H11ClN4
EXACTMASS=0.0
PEPMASS=223.0746
ADDUCT=M+H
56.049587 1795663.0
63.023022 239127.875
64.018227 69370.1328125
65.038643 25489.291015625
72.984047 933191.5625
72.985107 18297.458984375
90.033775 287269.75
91.04158 150971.84375
98.999519 450908.09375
99.001572 18864.412109375
126.010483 4403412.5
144.02121 23921.70703125
155.036865 15769.90234375
181.052856 78094.0
187.097778 65660.1484375
196.06369 24316.8125
202.077698 382861.25
203.080627 24936.255859375
203.085693 42338.2734375
206.048233 37634.5078125
223.074509 1126511.375
END IONS


BEGIN IONS
NAME=Amoxicillin
```

```
INCHIAUX=
MSLEVEL=MS2
INSTRUMENT_TYPE=Orbitrap
SOURCE_INSTRUMENT=
IONMODE=Positive
COLLISION_ENERGY=
FORMULA=C16H19N3O5S
EXACTMASS=0.0
PEPMASS=366.112
ADDUCT=M+H
53.038818 1134.14892578125
70.065269 1489.6866455078125
85.039719 3375.847412109375
87.026268 1992.7305908203125
100.392601 1055.7734375
113.034485 11849.470703125
114.037086 25466.88671875
160.033981 2116.989501953125
160.04274 78395.5390625
162.980865 1001.30859375
202.077698 22082.623046875
203.083862 3680.696533203125
207.076645 1640.1524658203125
268.052948 1057.2850341796875
END IONS


BEGIN IONS
NAME=Aripiprazole
INCHIAUX=
MSLEVEL=MS2
INSTRUMENT_TYPE=Orbitrap
SOURCE_INSTRUMENT=
IONMODE=Positive
Collision energy=
FORMULA=C23H27Cl2N3O2
EXACTMASS=0.0
PEPMASS=448.1557
ADDUCT=M+H
56.049496 78281.9296875
65.038643 59905.984375
70.065208 67904.9765625
84.080719 45626.17578125
90.046288 48200.53125
91.054146 149250.28125
95.049011 79246.828125
98.096336 511891.75
117.057243 178648.453125
118.065079 44101.23828125
148.075775 124458.9453125
153.034012 44469.02734375
164.070648 236585.421875
172.966766 42473.58984375
176.070618 601961.1875
```

```
188.002914 101346.1015625
202.077698 289374.96875
218.117538 273376.96875
243.045288 94822.5625
285.092072 1879308.75
448.155731 1277690.125
END IONS


BEGIN IONS
NAME=Azithromycin
INCHIAUX=
MSLEVEL=MS2
INSTRUMENT_TYPE=Orbitrap
SOURCE_INSTRUMENT=
IONMODE=Positive
Collision energy=
FORMULA=C38H72N2O12
EXACTMASS=0.0
PEPMASS=749.5154
ADDUCT=M+H
81.069885 2990.516845703125
83.049141 163042.140625
84.080757 6544.45849609375
87.044022 3489.7802734375
88.07563 9400.2744140625
98.096298 35915.4375
99.08033 4168.9248046875
100.075569 3052.1982421875
113.059624 7619.75732421875
114.091408 2474.423095703125
116.070549 37162.8203125
116.106911 110392.046875
127.075195 4725.66259765625
156.138443 2783.52392578125
158.117645 126806.5546875
186.148743 6271.7802734375
202.077698 26009.291015625
203.080765 2413.98681640625
203.085556 2965.19580078125
398.290283 8089.7587890625
416.301239 5091.197265625
434.311493 15966.5546875
457.327026 2749.672119140625
573.411133 38121.9921875
591.421875 101131.0390625
592.428894 4056.916015625
749.515564 8515.87109375
END IONS


BEGIN IONS
NAME=Benzophenone
INCHIAUX=
```

MSLEVEL=MS2
INSTRUMENT_TYPE=Orbitrap
SOURCE_INSTRUMENT=
IONMODE=Positive
Collision energy=
FORMULA=C13H10O
EXACTMASS=0.0
PEPMASS=183.0804
ADDUCT=M+H
51.023022 53790.5703125
53.038647 3395.28857421875
56.942333 7706.3017578125
58.862827 2645.631591796875
59.049198 2737.052490234375
67.05442 3492.25244140625
72.937263 5070.16748046875
77.038628 56256.68359375
95.049088 115571.5546875
105.028824 6194.01025390625
105.033432 724829.25
105.044594 81996.2109375
105.046852 3487.287353515625
106.03669 4677.7421875
113.13607 2625.821044921875
113.963631 16421.974609375
131.974075 3565.01953125
132.95874 2961.178466796875
141.958466 2743.005615234375
159.969376 4488.140625
182.902756 2879.06494140625
183.080475 114401.9453125
183.124344 3729.366455078125
183.160614 3277.894775390625
202.077698 85105.09375
203.081009 6856.8203125
203.085632 7168.3095703125
203.513672 2602.034912109375
END IONS

BEGIN IONS
NAME=Bis(2-ethylhexyl) phthalate
INCHIAUX=
MSLEVEL=MS2
INSTRUMENT_TYPE=Orbitrap
SOURCE_INSTRUMENT=
IONMODE=Positive
Collision energy=
FORMULA=C24H38O4
EXACTMASS=0.0
PEPMASS=391.2847
ADDUCT=M+H
57.069977 47848.21875
65.038689 76578.359375

71.085617 76208.7265625
149.023361 722478.125
202.07164 24995.623046875
202.077698 622448.75
203.080902 49424.23828125
203.085907 38274.640625
END IONS

BEGIN IONS
NAME=BleomycinA5
INCHIAUX=
MSLEVEL=MS2
INSTRUMENT_TYPE=Orbitrap
SOURCE_INSTRUMENT=
IONMODE=Positive
COLLISION_ENERGY=
FORMULA=C57H89N19O21S2
EXACTMASS=0.0
PEPMASS=751.2608
ADDUCT=M+1
182.340866 1619.6173095703125
202.077698 51868.171875
203.084534 8361.998046875
260.79483 1716.8802490234375
264.722565 1882.7364501953125
281.313232 1941.8582763671875
341.180389 1767.151611328125
345.410919 1597.6229248046875
END IONS

BEGIN IONS
NAME=Bromazepam
INCHIAUX=
MSLEVEL=MS2
INSTRUMENT_TYPE=Orbitrap
SOURCE_INSTRUMENT=
IONMODE=Positive
Collision energy=
FORMULA=C14H10BrN3O
EXACTMASS=0.0
PEPMASS=316.0081
ADDUCT=M+H
51.023022 23116.517578125
76.030823 17286.90234375
77.03862 13578.6181640625
78.033821 37685.4921875
78.046417 37704.109375
79.041664 24985.361328125
80.049477 148591.296875
89.038559 16025.7890625
95.049042 44596.08984375
96.044273 69265.1875

```
103.041481 16672.921875
104.049355 96784.6015625
105.044586 41695.05078125
105.057129 12169.6259765625
124.039307 21373.52734375
126.046265 12348.4345703125
127.054214 28168.431640625
128.049393 27808.91015625
129.044662 15782.62890625
129.057327 13371.642578125
130.065125 30881.748046875
133.052185 23315.55078125
140.04953 26447.5625
145.064911 19116.28515625
146.06012 12327.86328125
152.049545 10625.3046875
153.057358 31949.6171875
154.065186 137412.0625
155.06041 51795.390625
164.049591 19788.033203125
165.057297 10322.82421875
179.060364 116374.4375
180.068115 41574.4296875
181.075974 91612.9609375
182.083832 576347.375
183.975571 173892.0625
192.068161 16465.828125
202.077698 42829.1875
206.071243 13866.845703125
207.079102 18249.853515625
208.086853 102733.984375
208.970825 32307.998046875
209.094711 540146.5
210.07872 10605.833984375
211.970657 18155.33203125
258.98645 54012.66015625
259.994293 117141.2421875
261.002106 358955.6875
288.013184 468696.375
315.983978 11978.6640625
315.999237 46826.95703125
316.008026 1924318.5
316.027252 12455.51171875
316.032776 12994.640625
END IONS


BEGIN IONS
NAME=Caffeine
INCHIAUX=
MSLEVEL=MS2
INSTRUMENT_TYPE=Orbitrap
SOURCE_INSTRUMENT=
IONMODE=Positive
```

```
Collision energy=
FORMULA=C8H10N4O2
EXACTMASS=0.0
PEPMASS=195.0876
ADDUCT=M+H
48.771488 13855.9150390625
48.772121 14060.7900390625
54.021366 26099.2890625
54.033875 16410.068359375
56.049496 83044.9765625
67.029175 24358.28125
68.036957 149400.140625
69.044807 101310.421875
81.044746 23155.150390625
83.060318 120783.328125
109.03933 18805.7421875
110.071121 291735.5625
123.042603 72808.3046875
138.059189 17986.302734375
138.066162 3036577.25
180.06398 15385.634765625
185.52211 14439.28515625
195.075684 39761.3203125
195.083115 86100.515625
195.08754 5782870.0
202.077698 174714.125
203.080582 18057.74609375
203.085815 24798.453125
END IONS


BEGIN IONS
NAME=Carbamazepine
INCHIAUX=
MSLEVEL=MS2
INSTRUMENT_TYPE=Orbitrap
SOURCE_INSTRUMENT=
IONMODE=Positive
Collision energy=
FORMULA=C15H12N2O
EXACTMASS=0.0
PEPMASS=237.1023
ADDUCT=M+H
65.038628 117471.15625
89.038422 330108.03125
90.046288 191364.796875
115.054199 146684.28125
139.054245 130764.515625
152.062057 490303.65625
153.057388 137931.625
164.062088 240763.8125
165.069855 1064716.25
166.065048 215396.546875
167.072891 423724.5625
```

```
169.064804 272195.71875
177.057266 148425.859375
178.065125 619061.375
179.060349 101017.0234375
179.072998 290672.5625
190.065048 447342.8125
191.072937 989934.875
192.080765 4083253.5
193.088593 710129.5625
194.096359 1.6533829E7
194.108185 118304.2734375
202.077698 946621.9375
203.08107 106158.9375
220.075623 255193.921875
237.102219 4676485.0
END IONS


BEGIN IONS
NAME=Chlormequat chloride
INCHIAUX=
MSLEVEL=MS2
INSTRUMENT_TYPE=Orbitrap
SOURCE_INSTRUMENT=
IONMODE=Positive
COLLISION_ENERGY=
FORMULA=C5H13ClN
EXACTMASS=122.073652
PEPMASS=122.0732
ADDUCT=M+
40.958717 225412.15625
42.033768 815759.0625
44.049473 392701.375
45.659763 206371.578125
49.331738 201226.578125
53.216263 182112.46875
53.969803 214539.84375
58.065212 4.4051112E7
59.071552 207814.859375
59.073021 1.0375188E7
60.080891 1264321.125
62.999672 7300466.0
63.001236 153650.59375
63.001812 298255.0
79.054337 336316.96875
80.694923 210910.640625
94.041641 386964.21875
94.381157 190079.75
94.932426 221092.625
102.771248 200403.703125
104.026039 302614.8125
105.044067 195292.75
105.319992 189765.921875
112.721764 193243.78125
```

122.044518 257737.625
122.054848 314575.875
122.061287 1433614.0
122.063744 1153705.125
122.073029 2.29884976E8
122.081985 1363328.125
122.084618 1636115.375
122.090828 348456.40625
122.096069 1.2300243E7
123.076294 224034.265625
127.393517 265138.34375
137.164047 216675.71875
137.932892 211608.265625
END IONS

BEGIN IONS
NAME=Ciprofloxacin
INCHIAUX=
MSLEVEL=MS2
INSTRUMENT_TYPE=Orbitrap
SOURCE_INSTRUMENT=
IONMODE=Positive
Collision energy=
FORMULA=C17H18FN3O3
EXACTMASS=0.0
PEPMASS=332.1408
ADDUCT=M+H
70.065224 29055.494140625
75.022964 17409.234375
89.038551 30852.728515625
107.02906 57086.73828125
128.049454 22002.58203125
129.044693 24766.578125
132.024338 24672.330078125
133.032227 37244.86328125
134.040085 34990.59375
135.047852 23722.849609375
148.055756 35928.71875
172.055695 18215.40234375
189.045822 22275.52734375
191.061447 36768.71484375
202.077698 101593.59375
203.061539 82766.046875
204.069244 126825.359375
205.077087 99856.3671875
217.076996 19284.736328125
219.092651 15836.1865234375
227.105225 16999.521484375
231.056396 105118.2109375
231.092728 101590.59375
245.108414 649995.1875
268.144409 103134.5078125
286.135406 33990.25

288.150757 946452.8125
312.134521 26450.513671875
314.129486 46925.578125
332.140686 1186515.875
END IONS

BEGIN IONS
NAME=Clarithomycin
INCHIAUX=
MSLEVEL=MS2
INSTRUMENT_TYPE=Orbitrap
SOURCE_INSTRUMENT=
IONMODE=Positive
Collision energy=
FORMULA=C38H69NO12
EXACTMASS=0.0
PEPMASS=748.4843
ADDUCT=M+H
79.054352 5234.95068359375
81.069954 14305.609375
83.049171 856263.125
84.052483 7805.7919921875
84.080788 32593.056640625
85.028343 6436.2373046875
87.044022 30862.95703125
88.075653 48930.9921875
95.049057 6932.837890625
98.096359 227633.828125
99.080383 9018.1376953125
99.104279 6774.3984375
100.075584 21158.24609375
113.059654 62259.421875
114.091263 18370.333984375
115.075302 12745.501953125
116.067833 5290.193359375
116.07058 250971.484375
116.104248 5048.7373046875
116.106949 238520.109375
123.080536 10287.2548828125
127.075455 28205.708984375
158.117691 1016304.375
158.126221 6869.90087890625
159.121124 11981.416015625
202.077698 76291.7265625
203.08107 8676.57421875
203.085846 9814.2353515625
558.36377 7518.25634765625
END IONS

BEGIN IONS
NAME=Codeine
INCHIAUX=

MSLEVEL=MS2
INSTRUMENT_TYPE=Orbitrap
SOURCE_INSTRUMENT=
IONMODE=Positive
Collision energy=
FORMULA=C18H21NO3
EXACTMASS=0.0
PEPMASS=300.1596
ADDUCT=M+H
58.065205 82092.8203125
77.038582 24753.896484375
95.049057 31957.390625
102.046204 25388.4296875
115.054115 215996.5
127.054176 48617.6875
128.061996 133387.4375
137.059723 26408.65234375
139.054199 57955.71875
141.069962 52313.16796875
145.064835 27735.71875
152.062103 243035.4375
153.069809 50117.13671875
155.049301 41233.96875
155.06041 35498.4375
161.059769 57108.24609375
164.062012 26908.98828125
165.06987 116699.0859375
169.064835 35376.64453125
175.075516 23188.875
181.064728 34949.25
183.080322 80657.1015625
187.075439 51742.75390625
189.069931 27014.888671875
191.085449 21203.98046875
193.064804 45544.46484375
199.07547 103168.859375
202.077698 184884.03125
203.080811 28340.06640625
203.085968 22155.984375
209.059769 27155.873046875
215.106583 223241.3125
225.090851 105214.71875
241.086014 56544.046875
243.101395 162094.390625
267.125275 23545.810546875
282.148834 85013.609375
300.159485 3359147.25
300.181854 23489.33984375
END IONS

BEGIN IONS
NAME=DEET
INCHIAUX=

MSLEVEL=MS2
INSTRUMENT_TYPE=Orbitrap
SOURCE_INSTRUMENT=
IONMODE=Positive
Collision energy=
FORMULA=C12H17NO
EXACTMASS=0.0
PEPMASS=192.1384
ADDUCT=M+H
44.013077 114360.4296875
59.992832 18557.92578125
63.022984 38309.859375
65.038651 1395569.125
65.039787 37263.4765625
72.043236 24991.380859375
72.044449 394504.03125
72.080864 49857.41015625
91.054146 1991374.375
91.056038 39255.12890625
95.049164 27685.625
100.075584 536769.75
105.044632 18533.970703125
109.064659 335769.65625
118.065002 40587.72265625
118.236298 17022.234375
119.049042 8838042.0
119.054543 59848.1640625
119.060127 176202.8125
120.052444 44389.42578125
123.43602 17902.232421875
139.083908 18025.12890625
192.123535 21117.666015625
192.138275 4975774.0
192.149307 21961.158203125
193.141907 39223.76171875
202.077698 336250.34375
203.080505 37218.50390625
203.085907 34318.8125
END IONS

BEGIN IONS
NAME=Diazepam
INCHIAUX=
MSLEVEL=MS2
INSTRUMENT_TYPE=Orbitrap
SOURCE_INSTRUMENT=
IONMODE=Positive
Collision energy=
FORMULA=C16H13ClN2O
EXACTMASS=0.0
PEPMASS=285.0791
ADDUCT=M+H
63.023083 128319.6171875

65.038673 360420.5
72.98407 100140.78125
77.038635 111370.453125
89.038536 1387293.0
90.046364 589586.625
91.054176 940831.5625
95.049011 330750.71875
98.99955 262168.53125
104.049416 152866.484375
105.033386 464125.78125
105.044586 189966.546875
116.049332 278605.53125
117.057259 368866.03125
118.065102 325536.1875
125.01532 248733.390625
138.010574 142682.59375
139.005814 102512.7265625
143.060471 233084.59375
152.06221 151681.859375
154.041916 2644992.0
154.045914 112615.03125
163.005661 154129.71875
165.06987 632537.875
167.013168 135892.078125
172.063324 278643.03125
179.037125 278202.5625
179.073135 314552.28125
180.021057 244480.40625
182.036758 634322.6875
192.080795 149015.59375
193.088608 1336537.25
202.077698 1913334.5
203.080887 232559.25
203.086243 100363.75
204.080704 119486.4375
205.075867 141323.734375
206.083969 217601.875
221.107376 160297.453125
222.115234 1378460.625
227.0495 152845.625
228.057495 1554585.875
230.072998 106215.40625
241.052673 294667.8125
242.061081 111191.21875
255.068451 214675.4375
257.084015 2391808.5
285.058258 195673.609375
285.079071 2.226841E7
285.099609 152030.984375
286.082764 126794.078125
END IONS

BEGIN IONS

```
NAME=Dibutyl decanedioate
INCHIAUX=
MSLEVEL=MS2
INSTRUMENT_TYPE=Orbitrap
SOURCE_INSTRUMENT=
IONMODE=Positive
Collision energy=
FORMULA=C18H34O4
EXACTMASS=0.0
PEPMASS=315.2531
ADDUCT=M+H
55.054256 27347.80859375
67.054306 20097.375
69.069931 36384.6015625
79.054253 22384.912109375
81.069855 38934.3515625
83.049103 10613.51953125
93.069908 28478.154296875
95.08548 11952.6044921875
97.064796 10612.32421875
97.101128 29229.662109375
107.085388 11370.0361328125
111.116737 20126.55859375
121.101105 114469.453125
139.111816 152818.09375
149.096039 14141.84765625
157.122513 15685.650390625
167.620956 10811.486328125
185.117264 40300.5859375
202.077698 289742.4375
203.080521 36171.66015625
203.086243 15346.650390625
203.127853 99587.890625
END IONS


BEGIN IONS
NAME=Diclofenac
INCHIAUX=
MSLEVEL=MS2
INSTRUMENT_TYPE=Orbitrap
SOURCE_INSTRUMENT=
IONMODE=Positive
Collision energy=
FORMULA=C14H11Cl2NO2
EXACTMASS=0.0
PEPMASS=296.0241
ADDUCT=M+H
150.046463 16845.265625
151.054214 14947.822265625
152.062027 17290.822265625
155.060349 4647.7373046875
169.064819 78223.109375
177.057297 53284.64453125
```

```
178.065094 54808.78125
179.06041 29116.5234375
179.072937 64350.24609375
202.077698 84157.3515625
203.08075 6482.09375
203.085953 7229.72412109375
214.041824 389724.375
215.049606 716185.1875
215.063141 4319.0224609375
216.052582 4528.80859375
250.018463 142791.984375
278.013336 8033.81640625
END IONS


BEGIN IONS
NAME=Dioxybenzone
INCHIAUX=
MSLEVEL=MS2
INSTRUMENT_TYPE=Orbitrap
SOURCE_INSTRUMENT=
IONMODE=Positive
Collision energy=
FORMULA=C14H12O4
EXACTMASS=0.0
PEPMASS=245.0809
ADDUCT=M+H
51.023029 8405.884765625
52.030834 35758.2734375
65.037567 7071.421875
65.038673 283715.78125
68.997208 10064.2265625
80.025642 10335.88671875
93.033401 15242.3701171875
95.049057 37055.71484375
108.020615 16534.9296875
111.044037 6783.849609375
121.022514 12299.6240234375
121.028343 1800521.0
121.039131 20517.0703125
122.031685 8246.662109375
139.039017 8730.8408203125
151.039047 649925.8125
151.041977 11928.693359375
169.04921 5331.36376953125
202.071655 5654.80029296875
202.077698 102897.671875
203.080811 12405.5576171875
203.085907 5318.02978515625
245.08078 161111.96875
END IONS


BEGIN IONS
```

NAME=Erythromycin
INCHIAUX=
MSLEVEL=MS2
INSTRUMENT_TYPE=Orbitrap
SOURCE_INSTRUMENT=
IONMODE=Positive
Collision energy=
FORMULA=C37H67NO13
EXACTMASS=0.0
PEPMASS=734.4688
ADDUCT=M+H
79.054314 41722.01171875
80.033768 11285.798828125
81.069939 31439.462890625
83.049187 1957311.375
84.052635 11366.025390625
84.080803 95725.828125
86.060211 12012.025390625
87.044098 64850.25390625
88.008667 10781.5185546875
88.075661 93234.765625
95.049133 14034.7060546875
97.064751 24951.76953125
98.094315 23472.18359375
98.096359 456741.0625
99.080292 26905.15234375
100.075661 43901.44921875
109.064751 16872.08984375
110.55542 13431.6943359375
113.059624 110121.7109375
114.091362 44282.71875
115.075432 23456.87890625
116.070587 599480.125
116.07325 18283.87109375
116.106949 597624.625
116.109619 13029.7744140625
122.191635 10937.177734375
123.080452 82095.703125
125.096283 10546.1220703125
127.075455 96739.9453125
139.362015 10087.0556640625
142.943024 10273.697265625
158.117722 2533032.5
159.120972 14494.9091796875
195.653885 10720.1064453125
199.732407 10156.173828125
202.077698 331018.90625
203.081085 41230.203125
203.085709 44532.8828125
207.015839 11694.7822265625
216.275604 10161.9658203125
233.153564 28279.056640625
342.227325 15642.732421875
347.2211 12530.6484375

367.953186 10305.4931640625
457.892578 10884.1884765625
558.362793 14902.76171875
576.373108 51634.953125
702.598755 10874.6259765625
END IONS

BEGIN IONS
NAME=Eusolex 6300
INCHIAUX=
MSLEVEL=MS2
INSTRUMENT_TYPE=Orbitrap
SOURCE_INSTRUMENT=
IONMODE=Positive
Collision energy=
FORMULA=C18H22O
EXACTMASS=0.0
PEPMASS=255.1745
ADDUCT=M+H
65.038689 27820.05859375
77.03862 23747.986328125
79.054237 31263.943359375
91.054161 58925.63671875
95.049049 32457.607421875
97.064697 123943.7578125
105.044579 29279.103515625
105.069771 119086.328125
111.080322 85464.453125
115.054138 46848.87109375
119.049049 31111.470703125
119.08548 58074.17578125
128.062073 36780.3125
129.069931 20400.193359375
131.085587 27446.20703125
141.069977 34473.77734375
143.085556 34838.890625
145.101196 46336.61328125
152.062134 22480.080078125
153.070084 20334.197265625
157.101212 123704.0625
159.116898 23210.064453125
163.111755 26293.896484375
165.069901 35875.16015625
169.101303 21617.91796875
171.08049 32263.669921875
171.116882 94408.296875
178.077789 25151.015625
183.116867 49327.3125
185.096069 36904.3046875
195.116791 80280.46875
197.132385 40411.48046875
199.111725 56777.3203125
202.077698 80439.0

212.119598 125200.140625
213.127457 72209.703125
237.163849 55709.85546875
255.174393 198696.703125
END IONS

BEGIN IONS
NAME=Ipconazole
INCHIAUX=
MSLEVEL=MS2
INSTRUMENT_TYPE=Orbitrap
SOURCE_INSTRUMENT=
IONMODE=Positive
Collision energy=
FORMULA=C18H24ClN3O
EXACTMASS=0.0
PEPMASS=334.1684
ADDUCT=M+H
63.023041 49534.34375
67.054367 34344.28125
70.037491 70149.2109375
70.040077 9601749.0
70.042038 60094.84375
70.04261 52273.27734375
72.984116 21668.828125
89.038536 185715.25
90.046379 95335.6015625
95.049141 27819.578125
95.085449 26330.546875
98.999519 117598.390625
109.101067 93060.3203125
115.054169 48720.83203125
125.015259 554132.875
128.061981 24433.169921875
141.070023 24329.09765625
151.030991 41949.46484375
163.031052 30271.947265625
177.046448 34667.36328125
191.062424 86431.6328125
202.077698 302520.90625
203.08107 42726.25390625
203.086029 27200.580078125
247.125153 21290.44921875
334.155212 25840.419921875
334.168335 845870.4375
334.181549 23083.431640625
END IONS

BEGIN IONS
NAME=Lorazepam
INCHIAUX=
MSLEVEL=MS2

INSTRUMENT_TYPE=Orbitrap
SOURCE_INSTRUMENT=
IONMODE=Positive
Collision energy=
FORMULA=C15H10Cl2N2O2
EXACTMASS=0.0
PEPMASS=321.0195
ADDUCT=M+H
51.023022 32207.173828125
65.038628 19799.96875
66.046494 73750.4921875
75.022987 137569.109375
76.018211 27773.31640625
91.041542 29033.478515625
93.057251 81047.5546875
100.018044 82595.65625
102.033707 74452.171875
120.044365 19010.794921875
128.026077 42481.69140625
130.039993 57469.66015625
138.010574 88461.5703125
139.005753 19772.8203125
139.054169 20648.34765625
150.046478 41943.4296875
163.005859 68706.5078125
166.005386 23363.916015625
166.065109 38899.734375
167.072815 20676.974609375
169.064819 26982.0859375
177.05748 47540.1328125
192.068283 31271.611328125
193.076035 50016.25
194.083725 19241.916015625
202.077698 217764.984375
203.080811 29951.119140625
229.052628 561311.25
239.037109 21745.271484375
250.018478 19270.443359375
265.029266 90949.09375
275.013824 2366404.25
303.008759 758835.4375
321.019318 249009.59375
END IONS

BEGIN IONS
NAME=MDMA
INCHIAUX=
MSLEVEL=MS2
INSTRUMENT_TYPE=Orbitrap
SOURCE_INSTRUMENT=
IONMODE=Positive
Collision energy=
FORMULA=C11H15NO2

EXACTMASS=0.0
PEPMASS=194.1176
ADDUCT=M+H
51.023006 190200.515625
55.017864 34957.7578125
58.065216 555112.0625
65.038696 160900.921875
77.038612 305727.0625
79.054268 417654.09375
91.054153 22664.076171875
95.049065 568838.5625
103.054184 187390.484375
105.033463 25379.720703125
105.044655 411602.625
105.069794 1089471.0
121.028511 33066.1796875
122.036201 30951.802734375
133.061539 32563.150390625
133.064819 1273425.875
135.044083 1434074.0
151.075378 99894.625
163.075424 4804493.5
163.08432 33953.07421875
194.117523 327278.9375
202.077698 258749.625
203.08078 21987.10546875
END IONS

BEGIN IONS
NAME=Meloxicam
INCHIAUX=
MSLEVEL=MS2
INSTRUMENT_TYPE=Orbitrap
SOURCE_INSTRUMENT=
IONMODE=Positive
Collision energy=
FORMULA=C14H13N3O4S2
EXACTMASS=0.0
PEPMASS=352.0422
ADDUCT=M+H
51.022995 25179.46484375
53.038685 28111.109375
56.049538 25065.677734375
58.995178 25216.48828125
59.990284 126586.140625
65.03862 70298.796875
66.046501 41449.04296875
69.044846 45687.31640625
70.028786 62962.10546875
71.990311 26385.19140625
73.010727 1174812.75
76.0308 21301.521484375
77.038597 26689.7109375

85.969475 34201.75
88.0215 169808.921875
94.041245 34953.7734375
95.04892 33022.44140625
97.010544 27948.263671875
113.016693 29078.298828125
115.027031 62614.23828125
115.028015 32121.197265625
115.032356 8897496.0
115.037628 72272.0
115.039169 25310.267578125
127.032341 37661.09375
137.005554 23547.939453125
141.011765 3374393.5
153.000473 116339.375
164.016327 42200.66015625
168.995422 23618.03515625
182.038361 31592.427734375
184.054031 124634.4609375
194.026871 73898.875
202.071686 21305.48046875
202.077698 508205.90625
203.081055 56710.49609375
203.085815 39133.00390625
210.021606 47089.7578125
265.01001 48701.1015625
352.042694 88893.4453125
END IONS

BEGIN IONS
NAME=Methotrexate
INCHIAUX=
MSLEVEL=MS2
INSTRUMENT_TYPE=Orbitrap
SOURCE_INSTRUMENT=
IONMODE=Positive
COLLISION_ENERGY=
FORMULA=C20H22N8O5
EXACTMASS=0.0
PEPMASS=455.1788
ADDUCT=M+H
52.018299 11554.2490234375
54.033978 15536.3857421875
65.038589 10306.9638671875
66.021294 10403.9609375
67.029114 8309.03515625
79.029076 22737.1875
79.054199 8220.90625
80.024323 17602.490234375
95.049057 14482.919921875
105.044609 10291.98828125
106.039825 37330.91015625
124.0504 11290.353515625

133.050888 14991.4140625
134.060089 348490.15625
135.063202 12834.080078125
175.072723 448578.375
176.081223 38400.8828125
177.088226 25752.328125
202.077698 81935.2421875
203.083862 8828.328125
308.125519 664248.875
309.128937 50987.78125
END IONS

BEGIN IONS
NAME=Morphine
INCHIAUX=
MSLEVEL=MS2
INSTRUMENT_TYPE=Orbitrap
SOURCE_INSTRUMENT=
IONMODE=Positive
COLLISION_ENERGY=
FORMULA=C17H19NO3
EXACTMASS=0.0
PEPMASS=286.1438
ADDUCT=M+H
58.065273 98685.5078125
65.038719 36290.859375
77.038628 21749.56640625
91.054207 83844.1953125
95.049004 61085.80859375
102.046478 24254.53125
103.054123 37038.50390625
105.044563 33748.55078125
115.054115 216216.046875
127.054352 60853.97265625
128.062042 247257.203125
131.048828 22879.55078125
139.054321 66826.9296875
141.06958 28551.130859375
145.065033 47161.15234375
147.044189 27739.92578125
152.062164 368948.375
152.069382 31856.3671875
153.070053 55333.8203125
155.060272 34938.3828125
155.085205 36405.26953125
157.065109 40624.13671875
164.062286 21585.904296875
165.069962 165174.09375
169.064804 83727.5625
173.059738 56740.24609375
173.096039 30808.99609375
179.060532 22259.466796875
181.064926 52424.7265625

```
183.08049 79730.7890625
185.060074 100283.4375
191.085831 29092.9296875
193.064926 38465.40625
199.075211 20372.802734375
201.091141 256295.109375
202.077698 511917.1875
203.08287 83253.8515625
209.060028 36181.5703125
211.075653 97966.125
219.080566 35728.5859375
227.070053 30739.681640625
229.085709 183023.640625
237.091354 21145.84765625
268.133667 65492.41015625
286.10199 28192.904296875
286.143982 5434780.5
287.147278 28096.56640625
END IONS


BEGIN IONS
NAME=Nicotine
INCHIAUX=
MSLEVEL=MS2
INSTRUMENT_TYPE=Orbitrap
SOURCE_INSTRUMENT=
IONMODE=Positive
COLLISION_ENERGY=
FORMULA=C10H14N2
EXACTMASS=0.0
PEPMASS=163.123
ADDUCT=M+H
52.037685 2147.76318359375
56.96484 48833.34765625
62.018509 17597.05859375
65.038773 2279.4013671875
80.04953 3644.68798828125
84.080925 3850.306884765625
84.956917 3712.93896484375
84.959656 191866.9375
89.038597 2466.837646484375
94.065018 2056.10546875
102.9701 32103.533203125
105.044609 2413.682373046875
106.064987 11070.1064453125
117.057106 8915.076171875
120.080841 4187.1748046875
120.981125 4761.47314453125
130.065125 19771.80859375
132.08075 33451.9609375
134.979065 6031.49267578125
143.001968 3012.298828125
152.989319 4777.1279296875
```

```
162.973572 8909.17578125
163.123047 36718.5078125
180.984283 10936.9072265625
END IONS


BEGIN IONS
NAME=Oxybenzone
INCHIAUX=
MSLEVEL=MS2
INSTRUMENT_TYPE=Orbitrap
SOURCE_INSTRUMENT=
IONMODE=Positive
Collision energy=
FORMULA=C14H12O3
EXACTMASS=0.0
PEPMASS=229.086
ADDUCT=M+H
50.015121 19067.275390625
51.023003 81831.8828125
52.030804 69666.6328125
65.038643 41717.28515625
67.054321 6278.9443359375
68.997192 11367.0478515625
77.038597 80208.8984375
79.017838 5925.494140625
80.025658 19494.154296875
95.049026 188750.6875
98.036133 11705.232421875
105.033386 779845.375
105.044548 100196.0859375
108.020424 28934.1015625
139.054214 18348.9375
151.039017 1570807.5
202.077698 91038.984375
203.080872 8336.701171875
203.085999 5519.58740234375
229.085846 636692.3125
END IONS


BEGIN IONS
NAME=Paraquat dichloride
INCHIAUX=
MSLEVEL=MS2
INSTRUMENT_TYPE=Orbitrap
SOURCE_INSTRUMENT=
IONMODE=Positive
COLLISION_ENERGY=
FORMULA=C12H14N2x2Cl
EXACTMASS=186.115698
PEPMASS=185.1073
ADDUCT=M-H
57.934887 29314.501953125
```

58.942657 10172.34765625
113.963486 7648.35888671875
115.054108 6514.48583984375
116.971809 22415.50390625
117.979698 55244.9296875
139.987991 20682.615234375
140.995804 37198.80078125
144.08078 7414.962890625
158.096817 9128.4775390625
170.083908 13765.85546875
171.091888 22665.541015625
184.985565 7772.66552734375
185.107315 76660.2890625
185.16481 5285.029296875
186.115219 6254.89697265625
202.077698 63944.7578125
203.08168 10694.0390625
END IONS

BEGIN IONS
NAME=Paraquat dichloride
INCHIAUX=
MSLEVEL=MS2
INSTRUMENT_TYPE=Orbitrap
SOURCE_INSTRUMENT=
IONMODE=Positive
COLLISION_ENERGY=
FORMULA=C12H14N2x2Cl
EXACTMASS=186.115698
PEPMASS=93.0572
ADDUCT=M
59.049297 2705.600341796875
65.038673 3307.337158203125
77.038795 4523.8154296875
85.539322 2973.425048828125
86.049446 3546.468994140625
89.038704 2645.918212890625
91.054146 2842.823974609375
92.049545 8266.84375
92.553337 7826.6064453125
93.045113 4323.8310546875
93.049461 15234.037109375
93.051064 12138.091796875
93.057243 2086579.125
93.063423 8740.634765625
93.065018 13768.4404296875
93.06723 6737.07275390625
93.555984 9874.7841796875
93.558868 187206.375
94.04673 14901.83984375
95.049004 12840.3583984375
102.046326 2648.59228515625
103.05423 10680.6982421875

104.049553 3709.13916015625
105.044586 13746.0888671875
105.554924 2948.018310546875
113.570374 7120.01513671875
115.0541 8382.365234375
116.049767 2911.51025390625
117.05751 2912.58349609375
118.064972 12548.2333984375
128.049606 8727.337890625
129.044998 3231.406982421875
129.057373 5319.42431640625
130.065247 8637.5390625
142.065399 13449.173828125
143.073044 7978.19921875
144.0811 20593.486328125
155.060486 19410.958984375
156.068649 5386.72216796875
158.096756 3932.754638671875
169.076141 26736.3515625
171.091827 88957.15625
172.094879 7159.64306640625
187.08696 3849.82568359375
202.077698 53669.19921875
203.083466 10243.845703125
END IONS

BEGIN IONS
NAME=Progesterone
INCHIAUX=
MSLEVEL=MS2
INSTRUMENT_TYPE=Orbitrap
SOURCE_INSTRUMENT=
IONMODE=Positive
Collision energy=
FORMULA=C21H30O2
EXACTMASS=0.0
PEPMASS=315.232
ADDUCT=M+H
65.038635 10389.9599609375
67.054352 11659.3662109375
79.054276 33799.28515625
81.069916 22756.80078125
83.049156 21567.849609375
85.064781 14969.8251953125
91.054184 22698.240234375
95.049088 23015.0078125
95.085472 15130.8837890625
97.064705 425268.9375
105.044579 13336.08984375
107.085495 10260.51171875
109.064713 376130.90625
123.080414 37352.5
145.101288 11685.7734375

147.116943 11578.1767578125
159.116898 12018.5458984375
173.132599 10934.990234375
202.077698 33440.5390625
255.210953 15329.587890625
279.211182 14408.291015625
297.221313 41091.5
315.231964 150605.875
END IONS

BEGIN IONS
NAME=Testosterone
INCHIAUX=
MSLEVEL=MS2
INSTRUMENT_TYPE=Orbitrap
SOURCE_INSTRUMENT=
IONMODE=Positive
Collision energy=
FORMULA=C19H28O2
EXACTMASS=0.0
PEPMASS=289.2163
ADDUCT=M+H
53.038631 37206.171875
55.017849 43512.74609375
55.05423 32641.91015625
65.038689 65946.828125
67.054329 51002.890625
77.038612 51288.98828125
79.05423 187652.921875
81.069862 93021.7421875
83.04911 93711.3984375
91.054146 106667.9609375
93.069771 66515.1875
95.049042 92624.1953125
95.085449 61106.84375
97.064667 1663379.125
105.044579 63692.703125
105.069725 44577.75
107.085419 39237.01171875
109.062881 32665.919921875
109.064667 1357411.5
119.085358 30333.56640625
121.101059 38130.578125
123.080353 109384.078125
133.101181 29587.197265625
145.101212 32766.80859375
147.116821 40902.6328125
159.116791 42938.47265625
161.132538 36465.24609375
171.116943 28962.0234375
175.148087 31791.51953125
177.127441 26830.005859375
187.148087 46940.99609375

199.148041 27532.419921875
202.077698 128274.15625
253.194962 120669.078125
271.205627 126312.0703125
289.216278 834286.1875
END IONS

BEGIN IONS
NAME=Tonalide
INCHIAUX=
MSLEVEL=MS2
INSTRUMENT_TYPE=Orbitrap
SOURCE_INSTRUMENT=
IONMODE=Positive
Collision energy=
FORMULA=C18H26O
EXACTMASS=0.0
PEPMASS=259.2056
ADDUCT=M+H
78.046356 17687.572265625
91.054176 43822.71484375
95.049057 14635.060546875
103.054169 14803.5615234375
104.062004 12874.873046875
105.06971 13494.2275390625
115.054131 13448.98046875
117.069824 17956.2109375
119.085426 23164.775390625
128.061966 12530.00390625
133.101151 85138.8203125
147.080536 104665.6875
161.096115 150590.125
175.101868 12527.076171875
175.111725 1912021.875
176.115158 20779.916015625
189.12738 47819.515625
202.077698 165721.203125
203.080841 19604.05078125
203.085709 13301.611328125
203.143021 25378.51171875
259.205566 390459.5625
END IONS

BEGIN IONS
NAME=Tris(1,3-dichloro-2-propyl) phosphate - TDCPP
INCHIAUX=
MSLEVEL=MS2
INSTRUMENT_TYPE=Orbitrap
SOURCE_INSTRUMENT=
IONMODE=Positive
Collision energy=
FORMULA=C9H15Cl6O4P

EXACTMASS=0.0
PEPMASS=428.8915
ADDUCT=M+H
74.999657 39021.33984375
98.984047 343674.1875
98.988327 2692.849853515625
140.010651 3595.327392578125
157.02597 3838.59521484375
202.077698 16542.2265625
203.080917 2494.376708984375
208.953171 21823.15234375
END IONS

BEGIN IONS
NAME=Tris(2,3-dibromopropyl) phosphate - TDBPP
INCHIAUX=
MSLEVEL=MS2
INSTRUMENT_TYPE=Orbitrap
SOURCE_INSTRUMENT=
IONMODE=Positive
COLLISION_ENERGY=
FORMULA=C9H15Br6O4P
EXACTMASS=0.0
PEPMASS=692.5886
ADDUCT=M+H
85.402245 1612.3265380859375
97.879211 1748.310302734375
98.984077 24582.02734375
105.081444 2020.8858642578125
113.615494 2002.2083740234375
118.948975 4872.66943359375
120.397163 1745.252685546875
134.444031 1844.5997314453125
170.534134 1698.18212890625
171.28569 1673.315185546875
202.077698 78365.2578125
203.08197 11723.9130859375
239.989273 1686.1187744140625
241.266312 1561.926513671875
245.810684 1571.7542724609375
250.658997 1806.18603515625
275.643066 1601.5465087890625
276.309296 1645.60498046875
316.692902 1733.519287109375
END IONS

BEGIN IONS
NAME=Zolpidem Carboxylic Acid
INCHIAUX=
MSLEVEL=MS2
INSTRUMENT_TYPE=Orbitrap
SOURCE_INSTRUMENT=

IONMODE=Positive
Collision energy=
FORMULA=C19H19N3O3
EXACTMASS=0.0
PEPMASS=338.1502
ADDUCT=M+H
65.037567 146943.328125
65.038681 4634441.0
66.046509 341915.25
67.04184 101811.9609375
82.933899 90305.59375
89.038521 177812.40625
90.022949 93458.2890625
91.054138 184601.453125
92.049416 1254211.375
93.05719 712279.625
94.253944 95864.7578125
103.054115 152293.5625
110.059952 1572853.75
116.049324 132934.328125
117.057243 135802.6875
118.064995 406022.90625
143.060638 139290.9375
145.075928 253781.21875
177.057449 139063.671875
191.073349 92628.4296875
202.077698 3257962.5
203.080536 453480.125
203.0858 254081.9375
205.076111 634108.9375
217.076218 116093.2421875
218.083649 628001.875
219.091644 2851798.25
220.099564 347784.34375
221.107391 884975.9375
249.102417 227804.484375
265.087494 92849.4375
265.097198 6884621.0
266.105072 8185698.5
266.114594 153819.671875
293.092255 8780178.0
338.150208 4.1353048E7
339.153473 360808.65625
END IONS
