# Supplementary material for: Nontargeted Urinary Profiling Strategy for Endocrine-Disrupting Chemicals in Women with Ovarian Malignancies
Source: Environ Sci Technol. 2025 Apr 22;59(17):8380–90. doi: 10.1021/acs.est.4c13290 (PMC12060279; doi:10.1021/acs.est.4c13290)
Supplement: Supplementary file 4 — es4c13290_si_004.pdf [file es4c13290_si_004.pdf]

```
BEGIN IONS
NAME=11-Nor-9-carboxy-thc
INCHIAUX=
MSLEVEL=MS2
INSTRUMENT_TYPE=Orbitrap
SOURCE_INSTRUMENT=
IONMODE=Negative
Collision energy=
FORMULA=C21H28O4
EXACTMASS=0.0
PEPMASS=343.1913
ADDUCT=M-H
65.039673 8870.427734375
67.018967 4783.0625
79.055283 3287.651123046875
106.042351 3174.076904296875
107.050156 6691.220703125
122.037346 6205.234375
135.117996 6144.89794921875
136.052979 16483.759765625
137.097244 7477.25732421875
148.053101 7255.11083984375
159.081543 11282.2744140625
161.09726 13747.1708984375
178.099899 5619.44921875
179.107758 45440.92578125
191.107819 89599.7109375
192.115662 9412.6044921875
202.078796 24207.62890625
203.082031 4054.74755859375
203.107849 15284.875
204.115616 6202.5751953125
205.123199 3330.004638671875
216.11554 10251.0126953125
229.123459 14140.1083984375
230.131241 6571.6552734375
231.139267 11040.9140625
245.154709 141987.71875
246.15802 5580.56298828125
255.139282 6565.564453125
267.139008 5191.88037109375
271.170563 12737.9892578125
281.191162 3073.20947265625
282.162415 3185.3876953125
297.186096 60613.9375
299.201691 626398.125
299.223633 4452.5810546875
300.205048 27013.796875
325.18103 89481.4765625
326.184875 3678.970947265625
343.191284 221427.21875
344.194672 10668.205078125
END IONS
```

```
BEGIN IONS
NAME=2,3,4,5-Tetrabromophenol
INCHIAUX=
MSLEVEL=MS2
INSTRUMENT_TYPE=Orbitrap
SOURCE_INSTRUMENT=
IONMODE=Negative
Collision energy=
FORMULA=C6H2Br4O
EXACTMASS=0.0
PEPMASS=404.6767
ADDUCT=M-H
78.915756 4956.57177734375
78.918869 625156.375
78.921341 4090.177001953125
78.921806 4354.69775390625
201.071091 2845.003662109375
202.078796 28081.171875
203.08223 3327.999755859375
324.75058 69708.9609375
404.676636 126518.15625
END IONS


BEGIN IONS
NAME=2,3,4,5-Tetrachlorophenol
INCHIAUX=
MSLEVEL=MS2
INSTRUMENT_TYPE=Orbitrap
SOURCE_INSTRUMENT=
IONMODE=Negative
Collision energy=
FORMULA=C6H2Cl4O
EXACTMASS=0.0
PEPMASS=228.8788
ADDUCT=M-H
59.517284 2933.564453125
61.947319 2804.86376953125
63.90543 3029.5419921875
67.196526 2788.8828125
79.643532 3033.810546875
83.041458 2917.073486328125
102.841858 2767.28662109375
124.694023 3027.307373046875
150.321106 3346.705322265625
150.728546 3072.643798828125
164.907196 15855.119140625
166.922638 2987.350830078125
183.049606 2772.940185546875
192.902054 487355.09375
193.905548 8066.2890625
202.078796 67674.4765625
203.082108 10333.2861328125
```

```
203.087265 6055.3955078125
228.878738 768534.625
229.88205 20723.935546875
END IONS


BEGIN IONS
NAME=2,4-Dibromophenol
INCHIAUX=
MSLEVEL=MS2
INSTRUMENT_TYPE=Orbitrap
SOURCE_INSTRUMENT=
IONMODE=Negative
Collision energy=
FORMULA=C6H4Br2O
EXACTMASS=0.0
PEPMASS=248.8557
ADDUCT=M-H
78.915779 2055.975341796875
78.918831 261184.875
78.921814 2077.07958984375
112.985657 1942.49462890625
168.929581 1854.65673828125
202.078796 11514.234375
203.082047 1779.757568359375
248.855576 84782.640625
249.858917 2372.09765625
END IONS


BEGIN IONS
NAME=2,4-Dichlorophenol
INCHIAUX=
MSLEVEL=MS2
INSTRUMENT_TYPE=Orbitrap
SOURCE_INSTRUMENT=
IONMODE=Negative
Collision energy=
FORMULA=C6H4Cl2O
EXACTMASS=0.0
PEPMASS=160.9567
ADDUCT=M-H
89.003227 2475.6220703125
104.986198 1915.228271484375
124.97998 18959.640625
132.981216 2451.698486328125
160.842041 22486.642578125
160.956635 91472.359375
161.021729 1114.6220703125
161.035599 1258.330322265625
161.959793 1062.275390625
END IONS
```

```
BEGIN IONS
NAME=2,4-Dihydroxybenzophenone
INCHIAUX=
MSLEVEL=MS2
INSTRUMENT_TYPE=Orbitrap
SOURCE_INSTRUMENT=
IONMODE=Negative
Collision energy=
FORMULA=C13H10O3
EXACTMASS=0.0
PEPMASS=213.0558
ADDUCT=M-H
65.003342 1038.0079345703125
65.039719 22529.314453125
75.02401 4067.80224609375
91.018883 1802.1109619140625
93.028488 1281.294189453125
93.029648 2263.978515625
93.030663 6991.4619140625
93.031418 3730.814697265625
93.034554 905721.1875
93.037605 6797.8701171875
93.038406 5550.1015625
93.039589 1998.6112060546875
94.03788 13760.3837890625
135.008713 2409.698974609375
141.894699 2086.87158203125
153.019684 1114.1617431640625
167.050278 2188.19482421875
169.065964 94743.6796875
170.069244 2562.767822265625
201.071198 1185.9261474609375
202.078796 16004.6318359375
203.082077 2221.944091796875
213.055756 34363.0546875
214.059235 1329.349609375
END IONS


BEGIN IONS
NAME=3,5,6-Trichloro-2-pyridinol
INCHIAUX=
MSLEVEL=MS2
INSTRUMENT_TYPE=Orbitrap
SOURCE_INSTRUMENT=
IONMODE=Negative
Collision energy=
FORMULA=C5H2Cl3NO
EXACTMASS=0.0
PEPMASS=195.913
ADDUCT=M-H
46.490284 5601.380859375
51.231094 6320.33349609375
53.923 5782.37939453125
```

64.648811 6888.8232421875
68.152077 6665.392578125
68.80883 7516.34619140625
71.170097 5671.63671875
72.728539 5710.923828125
81.284805 5490.15576171875
95.944023 6410.673828125
118.443176 6286.724609375
135.398315 6260.5595703125
137.820312 6394.921875
162.983292 6136.6875
176.797043 6185.11181640625
195.906998 10832.53515625
195.912918 713067.8125
196.916687 11283.53515625
201.070801 12818.75390625
202.078796 173176.171875
203.081955 25631.8359375
203.086884 15713.9248046875
206.012268 6434.7451171875
210.9561 6603.4287109375
END IONS

BEGIN IONS
NAME=3-Phenoxybenzoic acid
INCHIAUX=
MSLEVEL=MS2
INSTRUMENT_TYPE=Orbitrap
SOURCE_INSTRUMENT=
IONMODE=Negative
Collision energy=
FORMULA=C13H10O3
EXACTMASS=0.0
PEPMASS=213.0558
ADDUCT=M-H
49.008408 17253.123046875
53.263634 3564.295654296875
53.264332 4584.830078125
63.024044 9217.193359375
65.003334 144773.203125
67.018959 8878.59375
91.018906 502314.6875
92.022186 7723.51904296875
92.998169 4398.939453125
101.039673 4081.069580078125
107.013702 4370.17724609375
108.021606 3083.640625
110.702965 2832.106201171875
119.050323 4668.0966796875
121.029549 15628.162109375
135.008835 690569.125
136.012207 14907.423828125
141.070969 10292.04296875

143.050232 15500.5791015625
153.019379 221076.1875
154.022766 6171.65869140625
154.042465 3707.936279296875
169.065933 361306.65625
170.069107 11542.51171875
171.04512 15586.0537109375
201.070923 3178.6171875
201.524597 2734.693359375
202.078796 45299.26171875
203.082138 6810.103515625
203.087036 3717.77685546875
213.03479 3915.893798828125
213.042221 13328.6318359375
213.044998 8894.5712890625
213.055756 2037907.75
213.066147 12916.0966796875
213.069153 14244.4658203125
213.080399 3804.939453125
214.059082 90473.390625
END IONS

BEGIN IONS
NAME=4-Nitrophenol
INCHIAUX=
MSLEVEL=MS2
INSTRUMENT_TYPE=Orbitrap
SOURCE_INSTRUMENT=
IONMODE=Negative
Collision energy=
FORMULA=C6H5NO3
EXACTMASS=0.0
PEPMASS=138.0198
ADDUCT=M-H
43.699844 3175.52001953125
45.993526 358447.6875
68.014328 3462.9931640625
87.023605 3066.5703125
92.026718 55486.8515625
94.459732 3342.518310546875
104.088173 3040.365966796875
108.02169 1418969.625
109.025032 42339.8984375
118.629639 3026.32275390625
122.952644 3090.476318359375
138.008636 7158.49853515625
138.012558 29793.6171875
138.014236 22147.7109375
138.019653 4093829.5
138.025253 21966.38671875
138.026611 27275.728515625
138.028778 10865.3544921875
139.017258 5882.40576171875

```
139.023041 105618.7578125
157.746094 3023.142578125
END IONS


BEGIN IONS
NAME=5-fluorouracil
INCHIAUX=
MSLEVEL=MS2
INSTRUMENT_TYPE=Orbitrap
SOURCE_INSTRUMENT=
IONMODE=Negative
COLLISION_ENERGY=
FORMULA=C4H3FN2O2
EXACTMASS=130.017855
PEPMASS=129.0106
ADDUCT=M-H
41.588039 13113.990234375
41.998592 2176082.0
44.259281 13130.9423828125
48.768703 12011.470703125
54.779354 11183.5849609375
55.09285 12072.4208984375
55.988735 13362.58203125
58.009895 16052.87890625
58.202461 14271.763671875
58.993832 140689.515625
60.025452 39327.62109375
65.540428 15324.5048828125
76.359619 11800.87109375
78.351692 13283.1435546875
85.029732 14523.7255859375
85.132042 11589.29296875
102.667343 11502.9267578125
113.503288 11305.6328125
120.135567 17282.744140625
120.683235 14989.66796875
128.975815 13546.310546875
128.997955 38975.6796875
129.010605 4277471.5
129.022858 18827.203125
END IONS


BEGIN IONS
NAME=PubChemalpha-Hexabromocyclododecane
INCHIAUX=
MSLEVEL=MS2
INSTRUMENT_TYPE=Orbitrap
SOURCE_INSTRUMENT=
IONMODE=Negative
COLLISION_ENERGY=
FORMULA=C12H18Br6
EXACTMASS=0.0
```

PEPMASS=634.6436
ADDUCT=M+H
86.944557 1894.9779052734375
97.147842 1471.69677734375
174.260712 1658.0560302734375
176.617035 1909.47705078125
195.715515 1616.2052001953125
201.070908 3376.943603515625
202.078796 60603.7265625
203.083618 7389.53125
299.163147 1710.3114013671875
300.807495 1754.1282958984375
333.896576 2169.267333984375
378.430786 1630.3580322265625
514.617615 1672.3468017578125
END IONS

BEGIN IONS
NAME=Bentazone
INCHIAUX=
MSLEVEL=MS2
INSTRUMENT_TYPE=Orbitrap
SOURCE_INSTRUMENT=
IONMODE=Negative
Collision energy=
FORMULA=C10H12N2O3S
EXACTMASS=0.0
PEPMASS=239.0495
ADDUCT=M-H
53.171703 17521.93359375
54.870407 18554.0859375
61.302036 17714.982421875
62.817139 19111.3125
65.173035 17408.466796875
77.965485 40858.00390625
79.957298 151469.078125
101.277618 19408.876953125
103.271179 17748.802734375
117.045837 78987.484375
132.032928 1862462.0
133.036316 52794.9609375
133.040756 821101.1875
147.081543 56665.0
153.390503 17831.62890625
162.825439 21347.716796875
165.372406 17964.908203125
175.087646 1523099.125
176.091049 61513.203125
192.472107 21318.90234375
195.994705 181562.703125
197.002655 1637904.0
198.00592 43099.5390625
201.070862 27087.78515625

```
202.078796 400229.375
203.081818 21184.486328125
203.08696 37280.42578125
239.034149 35553.69140625
239.036316 20876.552734375
239.049576 6186466.5
239.065521 40886.39453125
240.053146 195843.171875
END IONS


BEGIN IONS
NAME=Bisphenol S
INCHIAUX=
MSLEVEL=MS2
INSTRUMENT_TYPE=Orbitrap
SOURCE_INSTRUMENT=
IONMODE=Negative
Collision energy=
FORMULA=C12H10O4S1
EXACTMASS=0.0
PEPMASS=249.0229
ADDUCT=M-H
62.256222 9355.5205078125
62.404507 5321.91064453125
63.434883 5051.20947265625
63.962494 27109.103515625
72.704071 5324.19921875
79.957291 5495.39599609375
92.025291 8226.98046875
92.026711 506638.875
93.030029 6005.17578125
93.034477 23741.73046875
108.02169 1247082.0
108.026489 7013.05712890625
109.024986 23541.560546875
117.034554 6184.396484375
139.993622 11831.5673828125
155.988693 596421.9375
156.992477 7942.38232421875
156.996628 10794.248046875
157.065994 16300.84765625
184.052979 81708.2421875
185.056107 8787.083984375
185.060837 86806.8515625
201.070953 13877.37890625
202.078796 103710.2578125
203.081726 9768.6748046875
203.086838 5751.900390625
222.963165 5103.44970703125
249.001266 6827.72900390625
249.00563 21037.564453125
249.022675 3279446.0
249.035767 14646.3505859375
```

249.039536 17299.7578125
249.044739 8022.87548828125
250.026169 94082.3046875
END IONS

BEGIN IONS
NAME=Butyl paraben
INCHIAUX=
MSLEVEL=MS2
INSTRUMENT_TYPE=Orbitrap
SOURCE_INSTRUMENT=
IONMODE=Negative
Collision energy=
FORMULA=C11H14O3
EXACTMASS=0.0
PEPMASS=193.087
ADDUCT=M-H
65.039703 4380.115234375
71.050247 9960.57421875
82.005913 3108.80859375
91.018875 8046.28173828125
92.02285 3624.666015625
92.026695 477588.875
92.030434 3283.779052734375
93.030449 8225.005859375
93.034508 190725.125
94.037888 3716.72314453125
95.01371 7089.86328125
108.021645 10714.814453125
119.013855 5030.0693359375
121.029495 4495.6103515625
136.016586 237918.25
137.020569 5935.94873046875
137.024399 189839.734375
138.027679 3898.796142578125
193.075302 3459.26904296875
193.077652 2661.460693359375
193.086945 554634.6875
193.096039 3560.824462890625
193.098434 3388.14306640625
194.090225 22906.91015625
202.078796 15824.5654296875
END IONS

BEGIN IONS
NAME=Cyclohexane-1,2-dicarboxylic acid, mono(7-hydroxy- 4-methyloctyl) ester - OH-MINCH
INCHIAUX=
MSLEVEL=MS2
INSTRUMENT_TYPE=Orbitrap
SOURCE_INSTRUMENT=
IONMODE=Negative

Collision energy=
FORMULA=C17H30O5
EXACTMASS=0.0
PEPMASS=313.2022
ADDUCT=M-H
107.050171 1357.612548828125
109.061211 1032.472412109375
109.065926 179313.5
109.069527 1367.29541015625
110.069298 3136.0810546875
125.060837 16001.330078125
153.045395 2455.280029296875
153.047516 4724.841796875
153.048904 1379.623291015625
153.055756 534211.0
153.06105 1762.2115478515625
153.06218 5009.8212890625
153.063751 2581.59326171875
154.059097 12692.1630859375
157.123367 3262.0419921875
159.139328 4108.61572265625
202.078796 15179.5810546875
203.082031 1032.458740234375
313.202118 5628.95458984375
313.238556 1903.68408203125
END IONS

BEGIN IONS
NAME=Cyclohexane-1,2-dicarboxylic acid, mono(7-hydroxy- 4-methyloctyl) ester - oxo-MINCH
INCHIAUX=
MSLEVEL=MS2
INSTRUMENT_TYPE=Orbitrap
SOURCE_INSTRUMENT=
IONMODE=Negative
Collision energy=
FORMULA=C17H28O5
EXACTMASS=0.0
PEPMASS=311.1866
ADDUCT=M-H
57.03463 2902.5712890625
79.957306 1298.087890625
109.064087 5038.2578125
109.065895 237628.90625
110.069237 2974.986328125
125.060799 26215.466796875
137.097183 1057.078125
153.036285 1038.58544921875
153.047592 4515.15869140625
153.052612 7845.54345703125
153.055725 621387.9375
154.05899 13590.87109375
155.107727 1295.990966796875

157.123444 3673.314697265625
174.955978 1829.3253173828125
197.102325 1016.3717651367188
201.071136 1660.537353515625
202.078796 23563.9921875
203.082108 3107.831787109375
203.087311 1243.8143310546875
257.236542 1097.9222412109375
310.871521 1020.7243041992188
311.168976 1157.59765625
311.186737 4311.5322265625
END IONS

BEGIN IONS
NAME=Cyclohexane-1,2-dicarboxylic acid, mono-(4-methyl octyl) ester - MINCH
INCHIAUX=
MSLEVEL=MS2
INSTRUMENT_TYPE=Orbitrap
SOURCE_INSTRUMENT=
IONMODE=Negative
Collision energy=
FORMULA=C17H30O4
EXACTMASS=0.0
PEPMASS=297.2072
ADDUCT=M-H
79.957314 7826.8671875
109.06591 92746.6953125
110.069199 2384.5634765625
119.050186 1962.7978515625
125.060829 8340.634765625
141.128448 3334.6826171875
153.05574 206948.1875
154.059113 5698.79443359375
183.012131 8747.458984375
202.078796 16248.05078125
203.081543 3005.990234375
203.087158 1177.0972900390625
297.153076 23831.115234375
297.207428 1070.558837890625
297.243652 1800.174560546875
298.156281 2293.01953125
END IONS

BEGIN IONS
NAME=Cyclohexane-1,2-dicarboxylic acid,mono-(7-carboxy- 4-methylheptyl) ester - cx-MINCH
INCHIAUX=
MSLEVEL=MS2
INSTRUMENT_TYPE=Orbitrap
SOURCE_INSTRUMENT=
IONMODE=Negative

Collision energy=
FORMULA=C17H28O6
EXACTMASS=0.0
PEPMASS=327.1815
ADDUCT=M-H
71.591545 1062.85009765625
73.86042 1225.342529296875
84.931244 1037.486083984375
90.701416 1101.8466796875
93.034691 1472.1209716796875
102.948738 3416.91552734375
109.06591 160582.75
110.069275 4204.9833984375
125.060829 18178.73828125
125.097206 1722.0594482421875
125.627182 1018.7799072265625
127.0765 18841.23828125
127.112885 33057.4921875
128.116104 1079.30615234375
131.26062 1010.9042358398438
134.881638 1105.7052001953125
137.097183 1850.6324462890625
144.230194 1017.5938720703125
146.938644 1886.01025390625
153.047623 2702.087646484375
153.055756 365837.6875
153.066269 1270.27490234375
154.058975 8171.3681640625
155.107925 3430.666748046875
158.319565 1058.5406494140625
171.066345 45085.3671875
171.102554 2640.3818359375
172.069748 1880.95703125
173.103256 1396.0538330078125
173.108459 5678.82861328125
173.118301 792060.625
173.131149 2517.210693359375
174.121613 18400.408203125
177.633713 1063.611572265625
201.070587 1233.3099365234375
202.078796 21232.87109375
203.082062 3607.2509765625
203.086914 2803.37109375
209.757767 1068.39892578125
236.343872 1112.954833984375
281.552185 1166.02880859375
311.734375 1096.58447265625
327.163422 2222.906494140625
327.18161 13089.1240234375
335.169189 1212.0906982421875
END IONS

BEGIN IONS

NAME=Diclofenac
INCHIAUX=
MSLEVEL=MS2
INSTRUMENT_TYPE=Orbitrap
SOURCE_INSTRUMENT=
IONMODE=Negative
Collision energy=
FORMULA=C14H11Cl2NO2
EXACTMASS=0.0
PEPMASS=294.0096
ADDUCT=M-H
50.334873 3343.209716796875
50.617451 3627.01953125
51.556927 3589.323486328125
53.107349 3189.90283203125
58.2272 3557.913330078125
60.057419 3372.593017578125
65.738892 3883.151123046875
67.376915 3367.040283203125
67.538925 4066.799560546875
69.243393 2948.6669921875
70.868744 3428.239501953125
72.956978 3218.674072265625
75.567299 3459.103515625
80.183601 3282.069091796875
92.261238 3509.918212890625
105.701736 3594.63525390625
112.030571 3824.53466796875
125.639702 4005.640625
154.751526 4103.71728515625
158.646637 3385.98046875
178.066284 5471.064453125
180.613266 3334.474609375
201.070877 6545.8828125
202.078796 119188.515625
203.082306 11840.8046875
214.04274 20280.46484375
235.110138 3862.28173828125
245.604919 3890.40185546875
250.019562 108996.6328125
253.904266 4110.99072265625
290.402863 3590.521484375
312.431244 3449.625732421875
END IONS

BEGIN IONS
NAME=Dioxybenzone
INCHIAUX=
MSLEVEL=MS2
INSTRUMENT_TYPE=Orbitrap
SOURCE_INSTRUMENT=
IONMODE=Negative
Collision energy=

FORMULA=C14H12O4
EXACTMASS=0.0
PEPMASS=243.0662
ADDUCT=M-H
65.039658 5539.53173828125
80.026657 3749.920166015625
93.034508 167560.78125
94.03788 4285.62451171875
108.021645 27110.984375
123.045143 129980.2578125
124.048286 2565.119873046875
146.961136 4930.4189453125
184.052933 5485.8955078125
195.044998 3266.474853515625
197.060638 2313.575927734375
199.076355 4024.035888671875
202.078796 12034.4462890625
210.032181 2006.262451171875
213.055679 2252.1015625
225.055725 3276.462646484375
243.066147 8547.068359375
END IONS

BEGIN IONS
NAME=Ethyl paraben
INCHIAUX=
MSLEVEL=MS2
INSTRUMENT_TYPE=Orbitrap
SOURCE_INSTRUMENT=
IONMODE=Negative
Collision energy=
FORMULA=C9H10O3
EXACTMASS=0.0
PEPMASS=165.0558
ADDUCT=M-H
41.003342 2796.78662109375
43.01899 5805.7822265625
65.039703 5604.39599609375
91.018867 6918.72314453125
92.022957 2958.953369140625
92.026703 522931.375
93.030457 4832.24267578125
93.034515 364954.3125
94.037895 3905.50830078125
95.013763 6987.32958984375
108.021927 6870.5673828125
119.013817 5122.4970703125
121.029449 4450.09033203125
125.873192 2131.6376953125
136.016602 241914.171875
136.932159 3204.43701171875
137.024399 405886.59375
138.027817 4787.6298828125

164.83609 6833.32275390625
165.041824 20426.3359375
165.046616 2647.7255859375
165.055679 555745.875
165.064865 4186.974609375
166.059097 7028.033203125
END IONS

BEGIN IONS
NAME=Fipronil sulfone
INCHIAUX=
MSLEVEL=MS2
INSTRUMENT_TYPE=Orbitrap
SOURCE_INSTRUMENT=
IONMODE=Negative
Collision energy=
FORMULA=C12H4Cl2F6N4O2S
EXACTMASS=0.0
PEPMASS=450.9265
ADDUCT=M-H
68.995781 243007.734375
74.003624 49072.51171875
82.960793 300240.40625
90.009636 66922.484375
98.003548 82716.9765625
122.003685 318415.5625
132.957718 244561.578125
148.006699 30423.7578125
183.017548 61883.171875
188.006577 70766.6171875
201.071213 46997.81640625
202.078796 666942.4375
203.082184 70770.3984375
203.087296 36279.875
208.012634 58096.9375
217.986755 58694.38671875
218.009827 260094.5
227.0177 33531.51171875
233.980743 36832.0078125
243.989502 925166.9375
246.015823 598826.125
261.986176 317150.34375
281.992615 6348036.5
282.996155 75513.7109375
300.991241 73670.1328125
345.954529 156630.9375
414.93161 59878.44921875
414.949677 2052111.5
414.967957 55290.19921875
END IONS

BEGIN IONS

```
NAME=Fipronil
INCHIAUX=
MSLEVEL=MS2
INSTRUMENT_TYPE=Orbitrap
SOURCE_INSTRUMENT=
IONMODE=Negative
Collision energy=
FORMULA=C12H4Cl2F6N4OS
EXACTMASS=0.0
PEPMASS=434.9315
ADDUCT=M-H
50.003689 35736.71484375
57.975754 155529.640625
74.003586 29567.1015625
81.975677 20577.22265625
86.003456 61463.203125
88.000397 34369.3828125
98.003716 44033.18359375
112.00045 62979.44921875
113.014572 45600.9140625
143.005219 52101.0390625
163.01152 43706.7109375
182.009781 119434.78125
183.017502 561177.9375
202.078796 358080.4375
203.082047 45463.89453125
203.086807 35682.50390625
212.949158 26636.095703125
216.97876 25172.677734375
217.986389 478864.875
242.984192 20351.0234375
243.98938 281256.46875
249.958496 1996668.0
250.96199 34368.28125
253.986633 46325.21484375
267.989563 136511.0625
277.953369 1190339.625
280.956787 68188.4140625
281.992493 341611.0625
287.961487 91946.9453125
317.969238 1013460.0
318.972565 27539.49609375
329.959625 1871149.625
330.963196 51995.6328125
350.988129 26950.6328125
365.936249 49873.87109375
END IONS


BEGIN IONS
NAME=Lorazepam
INCHIAUX=
MSLEVEL=MS2
INSTRUMENT_TYPE=Orbitrap
```

```
SOURCE_INSTRUMENT=
IONMODE=Negative
Collision energy=
FORMULA=C15H10Cl2N2O2
EXACTMASS=0.0
PEPMASS=319.0049
ADDUCT=M-H
102.034874 219944.40625
116.014175 5381.134765625
151.990891 63310.56640625
173.035751 6069.62646484375
179.00174 28006.017578125
179.98584 19283.2578125
200.027069 7414.30712890625
201.070633 5657.859375
202.078796 73279.328125
203.082062 11610.34765625
227.038239 13351.8916015625
228.022003 20105.13671875
253.017776 5148.01416015625
255.033173 50492.875
283.027954 568591.1875
284.031342 32508.890625
291.009521 5835.337890625
END IONS


BEGIN IONS
NAME=Malathion dicarboxylic Acid
INCHIAUX=
MSLEVEL=MS2
INSTRUMENT_TYPE=Orbitrap
SOURCE_INSTRUMENT=
IONMODE=Negative
Collision energy=
FORMULA=C6H11O6PS2
EXACTMASS=0.0
PEPMASS=272.9662
ADDUCT=M-H
140.978104 1085.4005126953125
156.955383 1114.4224853515625
201.071259 2152.138427734375
202.078796 16006.484375
203.082397 2071.969482421875
203.087372 1036.9598388671875
244.903702 1311.6646728515625
END IONS


BEGIN IONS
NAME=Meloxicam
INCHIAUX=
MSLEVEL=MS2
INSTRUMENT_TYPE=Orbitrap
```

SOURCE_INSTRUMENT=
IONMODE=Negative
Collision energy=
FORMULA=C14H13N3O4S2
EXACTMASS=0.0
PEPMASS=350.0273
ADDUCT=M-H
57.975754 6972.1181640625
65.014565 9825.1162109375
86.006958 4087.09423828125
91.055305 3572.020751953125
97.9944 13250.7314453125
106.996147 3097.173828125
113.017944 339295.78125
114.021294 4091.126220703125
119.050293 63530.5625
128.050583 5498.40283203125
131.03772 99613.7890625
134.00708 3877.103271484375
136.022552 8750.6279296875
144.045532 33065.00390625
145.029556 11815.4375
146.061188 532778.0
146.068787 3458.220947265625
147.064468 11473.0302734375
148.994095 36906.3125
150.985794 5560.18115234375
160.040451 3845.40283203125
164.0177 19539.39453125
176.989059 8699.1484375
182.039566 6659.40673828125
192.012527 73224.6484375
202.078796 27553.513671875
203.082123 3576.1513671875
208.088242 3356.062744140625
210.023071 53633.078125
252.077957 31836.916015625
271.042236 9255.076171875
286.065613 99428.6171875
287.069153 5128.43896484375
END IONS

BEGIN IONS
NAME=Methyl paraben
INCHIAUX=
MSLEVEL=MS2
INSTRUMENT_TYPE=Orbitrap
SOURCE_INSTRUMENT=
IONMODE=Negative
Collision energy=
FORMULA=C8H8O3
EXACTMASS=0.0
PEPMASS=151.0401

```
ADDUCT=M-H
41.003334 1440.2779541015625
63.003231 1102.0523681640625
82.005913 4433.744140625
91.018867 4988.74658203125
92.026711 535943.125
92.030487 3658.470703125
93.029984 4562.80029296875
93.034462 3994.758056640625
95.013763 8158.3056640625
106.993233 3084.03173828125
107.050224 2433.978515625
108.021721 10440.3349609375
119.013855 6271.3408203125
121.029541 3208.123046875
136.016602 314887.6875
136.023346 2271.419921875
137.019882 4057.592041015625
151.040085 496436.5
151.047928 2769.318115234375
151.050568 1490.3271484375
152.043289 5361.90966796875
END IONS


BEGIN IONS
NAME=Mono(2-ethyl-5-carboxypentyl) phthalate - cx-MEPP
INCHIAUX=
MSLEVEL=MS2
INSTRUMENT_TYPE=Orbitrap
SOURCE_INSTRUMENT=
IONMODE=Negative
Collision energy=
FORMULA=C16H20O6
EXACTMASS=0.0
PEPMASS=307.1188
ADDUCT=M-H
55.214878 3871.76513671875
63.714363 3516.734619140625
65.327942 3850.68994140625
66.840508 3453.344482421875
76.577126 3563.878173828125
81.656197 3562.781494140625
83.834686 3842.45751953125
90.340782 3366.0068359375
94.533272 3465.4208984375
97.165329 3367.89501953125
105.974945 3501.365234375
107.852325 3880.749755859375
111.081429 7432.5556640625
113.097229 180184.5625
115.112793 9993.1201171875
121.029564 116885.984375
123.081749 5046.9833984375
```

126.380745 3878.657470703125
138.130295 3464.2109375
141.092072 5169.12548828125
144.816833 3627.42822265625
147.559814 3487.123046875
157.086914 5605.90478515625
159.102692 810740.4375
160.106064 12729.2822265625
160.114761 3793.58251953125
165.019363 41592.234375
192.690979 3832.930908203125
201.071213 9103.1630859375
202.078796 81039.875
203.081955 10036.634765625
203.312851 3925.7021484375
220.510681 4208.19091796875
221.518692 3706.341552734375
259.179871 3923.4091796875
323.14209 4052.953125
328.811157 4703.05078125
END IONS

BEGIN IONS
NAME=Mono(2-ethyl-5-hydroxy-hexyl) phthalate - OH-MEHP
INCHIAUX=
MSLEVEL=MS2
INSTRUMENT_TYPE=Orbitrap
SOURCE_INSTRUMENT=
IONMODE=Negative
Collision energy=
FORMULA=C16H22O5
EXACTMASS=0.0
PEPMASS=293.1396
ADDUCT=M-H
57.034637 4083.921630859375
71.050316 3145.4716796875
75.024033 7279.53076171875
99.081482 13668.4775390625
101.097115 2688.93017578125
113.09729 6207.95849609375
121.02037 2160.05810546875
121.023895 4254.81298828125
121.025017 8282.9765625
121.029564 761177.1875
121.032578 4708.9716796875
121.035309 6216.33544921875
122.032867 19520.34375
127.112953 9602.59765625
143.104218 2368.86328125
143.107803 64073.65234375
145.123428 197228.90625
146.126801 5761.728515625
147.008835 12076.33203125

165.019516 3039.599853515625
201.070724 3275.413330078125
202.078796 38453.078125
203.082047 6067.84423828125
203.087173 2539.893798828125
293.139618 18408.71484375
END IONS

BEGIN IONS
NAME=Mono(2-ethyl-5-oxo-hexyl) phthalate - oxo-MEHP
INCHIAUX=
MSLEVEL=MS2
INSTRUMENT_TYPE=Orbitrap
SOURCE_INSTRUMENT=
IONMODE=Negative
Collision energy=
FORMULA=C16H20O5
EXACTMASS=0.0
PEPMASS=291.1238
ADDUCT=M-H
57.034573 46655.94140625
59.013878 8424.2978515625
69.034668 3676.883544921875
71.050217 65283.1171875
75.024002 6549.28369140625
83.050163 5123.55517578125
99.08149 52703.65234375
113.097198 85969.546875
113.099731 3211.440185546875
115.112823 6167.76416015625
119.050224 51752.76953125
121.023834 13285.279296875
121.024994 11167.9892578125
121.029495 1262839.25
122.032845 21884.845703125
125.097214 16780.5078125
143.107727 394594.8125
144.111099 9486.8681640625
147.008728 8663.2421875
165.019485 5129.7919921875
202.078796 52759.81640625
203.082214 6698.59423828125
203.087143 3035.5888671875
205.123337 8690.9267578125
291.123932 9969.4521484375
END IONS

BEGIN IONS
NAME=Mono-(4-methyl-7-carboxyheptyl) phthalate - cx-MINP
INCHIAUX=
MSLEVEL=MS2
INSTRUMENT_TYPE=Orbitrap

```
SOURCE_INSTRUMENT=
IONMODE=Negative
Collision energy=
FORMULA=C17H22O6
EXACTMASS=0.0
PEPMASS=321.1344
ADDUCT=M-H
66.323853 5592.62841796875
75.024002 11377.42578125
85.715172 5055.85693359375
93.079315 5383.38037109375
108.042145 5562.7548828125
121.02948 677715.9375
122.033081 8984.3984375
125.097176 15819.515625
127.112808 136588.84375
137.097183 5550.82568359375
147.008804 14196.8603515625
155.107544 13754.451171875
165.019272 75855.3515625
167.371353 5248.8291015625
171.102463 18943.439453125
173.11824 2204838.0
173.128052 20420.048828125
174.121597 73380.8203125
189.923157 6154.50390625
201.070862 21352.869140625
202.072922 9187.9130859375
202.078796 199827.421875
203.081589 33711.828125
203.086807 13851.451171875
226.729889 5357.69091796875
241.88829 5082.4580078125
269.774933 5418.79052734375
317.303131 5867.38037109375
330.863647 5361.85107421875
END IONS


BEGIN IONS
NAME=Mono-(4-methyl-7-oxooctyl) phthalate - oxo-MINP
INCHIAUX=
MSLEVEL=MS2
INSTRUMENT_TYPE=Orbitrap
SOURCE_INSTRUMENT=
IONMODE=Negative
Collision energy=
FORMULA=C17H22O5
EXACTMASS=0.0
PEPMASS=305.135
ADDUCT=M-H
57.034622 93510.4453125
69.034515 9918.552734375
71.050224 9456.4755859375
```

75.024002 28265.83203125
77.039719 10765.4833984375
93.034561 10223.244140625
111.081558 79255.15625
113.097229 19368.587890625
119.050201 79864.078125
121.020798 9163.30859375
121.023834 26620.158203125
121.026398 37176.1875
121.02951 3843843.25
121.03405 43306.81640625
121.035316 24363.515625
122.032906 60708.41796875
127.112877 63930.7734375
134.037277 24098.4921875
137.097229 66702.2421875
139.112854 23603.8828125
147.005127 10661.92578125
147.008713 69541.03125
155.107697 67253.5390625
157.123398 398238.75
201.070908 16708.443359375
202.078796 174214.359375
203.082138 20437.755859375
203.087097 13433.95703125
261.149597 27265.32421875
305.139435 84040.65625
END IONS

BEGIN IONS
NAME=Mono-(6-hydroxy-2-propylheptyl) phthalate - OH-MIDP
INCHIAUX=
MSLEVEL=MS2
INSTRUMENT_TYPE=Orbitrap
SOURCE_INSTRUMENT=
IONMODE=Negative
Collision energy=
FORMULA=C18H26O5
EXACTMASS=0.0
PEPMASS=321.1708
ADDUCT=M-H
57.034592 3767.707763671875
75.023964 5607.77685546875
102.94873 2360.894287109375
121.023911 5667.73388671875
121.026482 7176.9833984375
121.029488 731571.4375
121.034004 7751.81884765625
121.035347 5421.10888671875
122.032822 11057.6474609375
127.112831 6997.4296875
141.128647 4245.89794921875
146.961212 6405.65234375

147.008713 9335.5947265625
165.019333 2739.206787109375
171.139038 28438.453125
173.154633 49314.2265625
174.956085 6589.58544921875
202.078796 31035.740234375
203.081543 4719.1298828125
203.086823 2568.70703125
321.170715 20797.4453125
END IONS

BEGIN IONS
NAME=Mono-(6-oxo-2-propylheptyl) phthalate - oxo-MIDP
INCHIAUX=
MSLEVEL=MS2
INSTRUMENT_TYPE=Orbitrap
SOURCE_INSTRUMENT=
IONMODE=Negative
Collision energy=
FORMULA=C18H24O5
EXACTMASS=0.0
PEPMASS=319.1551
ADDUCT=M-H
57.034599 31308.951171875
57.035477 1464.222412109375
59.013866 2020.8240966796875
69.034599 1698.23388671875
71.050217 3069.6376953125
72.276184 1077.34814453125
75.024017 3569.73828125
85.065796 15321.5458984375
93.034569 1030.7510986328125
99.081573 4677.1181640625
119.050201 18278.658203125
121.017189 1170.2850341796875
121.020538 1478.6025390625
121.023903 4131.86376953125
121.025047 8972.08984375
121.029488 695626.5
121.035416 3182.824951171875
122.032845 19904.62109375
122.035545 1970.6536865234375
125.097084 10160.6044921875
127.112778 14640.1533203125
130.431824 1002.3019409179688
134.037292 2194.38671875
139.112778 1742.238525390625
141.128494 26942.728515625
142.132065 1121.0059814453125
143.14418 2799.928466796875
147.008682 5515.287109375
151.112823 8049.673828125
153.128387 5868.01611328125

165.019226 2213.723388671875
167.107498 2033.3870849609375
169.12326 6228.49609375
171.139023 142479.359375
172.142487 4761.19921875
202.078796 24436.5859375
203.081955 3353.37548828125
203.087204 1971.0745849609375
233.154495 2898.9638671875
244.757324 1011.8443603515625
275.164948 1075.50439453125
319.154968 10319.263671875
END IONS

BEGIN IONS
NAME=Mono-2-ethylhexyl phthalate MEHP
INCHIAUX=
MSLEVEL=MS2
INSTRUMENT_TYPE=Orbitrap
SOURCE_INSTRUMENT=
IONMODE=Negative
Collision energy=
FORMULA=C16H22O4
EXACTMASS=0.0
PEPMASS=277.1446
ADDUCT=M-H
75.024025 6670.599609375
93.034554 2582.066162109375
103.018967 1270.8040771484375
107.050209 9075.3125
121.029541 77604.9140625
122.032707 1701.56494140625
125.09716 1318.5299072265625
127.112885 103855.921875
128.115967 3011.079345703125
129.12854 5093.853515625
134.037369 192009.875
134.044083 1831.6666259765625
135.040833 5012.1484375
135.045151 9600.36328125
147.008774 18374.103515625
165.019287 8185.607421875
188.035217 1400.4373779296875
201.071106 1629.2181396484375
202.073029 1057.434326171875
202.078796 17255.49609375
203.082047 1881.117431640625
203.144211 2140.77099609375
205.15979 4831.02783203125
233.154617 2560.007568359375
277.144501 10377.544921875
277.183594 1191.5025634765625
END IONS

```
BEGIN IONS
NAME=Mono-benzyl phthalate MBzP
INCHIAUX=
MSLEVEL=MS2
INSTRUMENT_TYPE=Orbitrap
SOURCE_INSTRUMENT=
IONMODE=Negative
Collision energy=
FORMULA=C15H12O4
EXACTMASS=0.0
PEPMASS=255.0663
ADDUCT=M-H
51.932743 8011.28759765625
53.768208 8649.720703125
71.470314 8389.4169921875
73.739937 8931.658203125
75.023979 63889.4453125
75.201736 9556.00390625
77.039719 13339.544921875
81.037712 8564.697265625
93.034531 14419.4697265625
99.479027 8917.8046875
105.034508 241837.328125
107.047852 21222.841796875
107.048561 8325.48828125
107.050186 576178.0625
118.21994 8621.15234375
119.013664 20408.236328125
121.029396 70976.3046875
121.602325 8992.224609375
123.045128 60946.75390625
142.00296 8058.6171875
144.355881 9599.3701171875
147.008713 162538.171875
149.024353 23202.73046875
165.019196 9278.53125
181.065582 12531.0517578125
183.081375 142203.828125
188.035553 10267.8857421875
188.843872 10429.2763671875
188.9319 8846.3271484375
202.078796 205195.25
203.081833 29569.11328125
203.087051 14162.583984375
208.227539 9549.1103515625
209.728043 8387.5166015625
237.452667 9939.34375
255.066162 26513.21484375
END IONS


BEGIN IONS
```

NAME=Mono-ethyl phthalate MEP
INCHIAUX=
MSLEVEL=MS2
INSTRUMENT_TYPE=Orbitrap
SOURCE_INSTRUMENT=
IONMODE=Negative
Collision energy=
FORMULA=C10H10O4
EXACTMASS=0.0
PEPMASS=193.0506
ADDUCT=M-H
45.175083 3789.63623046875
45.657845 3971.433837890625
46.721596 3763.6748046875
49.299759 3708.23046875
51.701122 3785.626953125
53.27116 3467.365966796875
53.39505 3846.53955078125
54.774063 4156.00439453125
54.892876 3784.3916015625
59.371616 4124.5927734375
65.307388 3819.505615234375
67.310822 3728.311767578125
75.02404 5999.2236328125
82.961197 3743.154296875
90.627235 3473.833251953125
100.016624 3484.074462890625
105.034637 3722.785400390625
107.05027 11399.998046875
119.050262 16864.853515625
121.029541 149714.96875
121.065872 55494.7890625
121.824974 3738.13427734375
127.494637 4527.53515625
134.03743 12729.7021484375
137.32225 3303.489501953125
147.008865 20461.005859375
147.045212 5307.85009765625
149.060867 9007.6708984375
165.019409 6616.18603515625
168.45694 3945.6435546875
180.511169 3470.2138671875
190.990906 3614.27685546875
193.05072 16620.716796875
201.070984 6504.52880859375
202.078796 123254.390625
203.082123 13913.2216796875
203.087234 7865.19091796875
206.018494 3382.014404296875
212.097427 3138.68359375
END IONS

BEGIN IONS

```
NAME=Mono-hydroxy-isononyl phthalate - OH-MINP
INCHIAUX=
MSLEVEL=MS2
INSTRUMENT_TYPE=Orbitrap
SOURCE_INSTRUMENT=
IONMODE=Negative
Collision energy=
FORMULA=C17H24O5
EXACTMASS=0.0
PEPMASS=307.1552
ADDUCT=M-H
57.03466 3860.406494140625
75.023979 6796.88037109375
93.034447 2418.5849609375
111.081581 2850.889404296875
113.097183 5162.05322265625
121.022186 2973.612548828125
121.023735 7401.01025390625
121.024902 5769.0546875
121.029495 1034615.625
121.033989 5172.93701171875
121.035263 6611.79638671875
122.032845 24097.107421875
127.112946 2823.899169921875
143.107681 2442.125732421875
147.008743 17141.955078125
155.107758 3342.513427734375
157.123367 43299.78515625
159.139008 42564.4609375
165.019379 4482.9443359375
202.078796 24969.349609375
203.081772 2562.59423828125
307.15506 36908.1875
308.159058 2049.076416015625
END IONS


BEGIN IONS
NAME=Mono-iso-butyl phthalate MiBP
INCHIAUX=
MSLEVEL=MS2
INSTRUMENT_TYPE=Orbitrap
SOURCE_INSTRUMENT=
IONMODE=Negative
Collision energy=
FORMULA=C12H14O4
EXACTMASS=0.0
PEPMASS=221.0848
ADDUCT=M-H
59.819572 10760.5634765625
66.207436 10764.7607421875
69.228065 11329.1376953125
71.049156 16225.318359375
71.050232 687015.0
```

```
72.053619 14452.4013671875
73.065857 12295.3837890625
75.024033 28160.28125
91.759087 10224.677734375
105.034653 14974.46484375
107.050392 32294.5703125
119.014053 13813.509765625
121.029533 291408.0
121.032173 16082.6015625
134.034073 23060.23046875
134.037338 590658.75
135.040894 25125.81640625
135.045319 38752.71875
147.008743 128441.5703125
147.081421 21411.498046875
149.097321 42017.18359375
165.019577 24790.263671875
177.092117 32826.5546875
201.071136 27775.14453125
202.078796 290848.125
203.082092 42027.28515625
203.087219 21471.2734375
221.082062 53493.6796875
231.706696 10044.5791015625
END IONS


BEGIN IONS
NAME=Mono-methyl phthalate MMP
INCHIAUX=
MSLEVEL=MS2
INSTRUMENT_TYPE=Orbitrap
SOURCE_INSTRUMENT=
IONMODE=Negative
Collision energy=
FORMULA=C9H8O4
EXACTMASS=0.0
PEPMASS=179.035
ADDUCT=M-H
44.998184 5345.849609375
45.656094 1043.6048583984375
50.707207 1006.8529663085938
52.111053 1102.07421875
56.367172 1024.777587890625
64.755806 1031.12841796875
65.014511 1383.9554443359375
65.998543 2284.2421875
67.030159 1254.46630859375
67.537605 1019.21044921875
83.766319 1077.1346435546875
88.754791 1042.32275390625
90.998032 7116.6103515625
96.421013 1086.9154052734375
105.034416 1557.7869873046875
```

107.050179 55600.76171875
111.873413 1068.8426513671875
121.029518 2083.820556640625
133.029495 1110.4512939453125
134.967865 1068.0421142578125
134.987961 10494.43359375
135.045151 3716.116943359375
135.064499 1936.6875
136.05159 1263.5428466796875
147.00885 1384.7647705078125
158.95108 1444.6082763671875
164.034271 1369.2578125
178.815079 1559.2763671875
178.977783 1235.44140625
179.034821 5501.427734375
179.057755 2001.025146484375
180.281891 1098.045654296875
201.070938 2329.77099609375
202.078796 30046.26953125
203.081818 2571.614990234375
203.08696 2189.700927734375
END IONS

BEGIN IONS
NAME=Mono-n-butyl phthalate MnBP
INCHIAUX=
MSLEVEL=MS2
INSTRUMENT_TYPE=Orbitrap
SOURCE_INSTRUMENT=
IONMODE=Negative
Collision energy=
FORMULA=C12H14O4
EXACTMASS=0.0
PEPMASS=221.0819
ADDUCT=M-H
51.325687 10011.3330078125
60.975975 10970.8486328125
64.041061 10930.6865234375
69.034592 308276.65625
71.049187 12764.1904296875
71.050232 910128.875
72.053604 13090.650390625
75.02404 30856.779296875
89.721527 10151.673828125
100.843452 10023.326171875
105.034538 10052.27734375
107.050247 48159.6015625
112.288498 10816.6826171875
121.026756 13560.5185546875
121.029549 322720.625
127.258278 11771.8935546875
134.037399 149063.28125
134.607742 10110.658203125

135.04512 25531.1015625
147.008713 122154.921875
147.081421 48409.640625
149.097015 48634.2265625
166.004013 10142.4462890625
177.092133 38412.6953125
183.237137 11523.69921875
201.071045 30494.318359375
202.078796 284654.125
203.081863 45211.4140625
203.086914 26559.453125
214.16748 10600.3291015625
221.081757 54230.10546875
END IONS

BEGIN IONS
NAME=Mono-n-octylphthalate MnOP
INCHIAUX=
MSLEVEL=MS2
INSTRUMENT_TYPE=Orbitrap
SOURCE_INSTRUMENT=
IONMODE=Negative
Collision energy=
FORMULA=C16H22O4
EXACTMASS=0.0
PEPMASS=277.1446
ADDUCT=M-H
75.024017 3476.648681640625
93.034485 1665.98193359375
107.050232 1346.835693359375
121.029533 50276.80859375
123.081528 1414.88232421875
125.097198 27734.17578125
127.112854 103690.515625
128.116196 2790.460693359375
134.034042 1097.565185546875
134.037308 17926.62890625
135.045181 2223.191650390625
146.961212 1046.6776123046875
147.008713 9586.087890625
165.019318 3083.19189453125
201.070938 1011.39306640625
202.078796 17414.388671875
203.082123 1660.3427734375
203.087173 1059.6715087890625
203.144302 4700.18408203125
205.1595 2997.045654296875
231.139084 1701.93896484375
233.15477 3154.870849609375
277.144043 2836.081787109375
END IONS

BEGIN IONS
NAME=Monocyclohexyl phthalate MCHP
INCHIAUX=
MSLEVEL=MS2
INSTRUMENT_TYPE=Orbitrap
SOURCE_INSTRUMENT=
IONMODE=Negative
Collision energy=
FORMULA=C14H16O4
EXACTMASS=0.0
PEPMASS=247.0976
ADDUCT=M-H
53.456799 4496.9296875
54.675613 4602.109375
56.098644 4370.08203125
57.048939 4251.439453125
58.504616 4120.03271484375
61.702103 4428.46044921875
68.855415 4456.5478515625
69.034584 17215.615234375
75.023972 38592.6328125
93.034492 13703.28515625
95.050194 101739.984375
97.065834 1152154.625
98.069107 24816.744140625
99.081436 45963.96484375
103.018829 5491.474609375
119.013817 8007.0732421875
121.029503 344661.0625
122.032928 7237.93798828125
125.060783 5157.72509765625
147.008774 163561.5
148.012207 8311.9609375
164.905823 4170.7646484375
165.01947 26000.865234375
165.132584 4001.409423828125
175.112762 59940.390625
185.096939 5330.24853515625
188.035416 7428.87109375
195.912399 4670.0009765625
201.091797 8650.4482421875
202.078796 117384.3828125
203.082184 11777.03515625
203.107712 22949.224609375
247.097549 116344.890625
248.100815 9744.9453125
END IONS

BEGIN IONS
NAME=O,O-Dimethyldithiophosphate
INCHIAUX=
MSLEVEL=MS2
INSTRUMENT_TYPE=Orbitrap

```
SOURCE_INSTRUMENT=
IONMODE=Negative
Collision energy=
FORMULA=C2H7O2PS2
EXACTMASS=0.0
PEPMASS=156.9553
ADDUCT=M-H
46.99614 10002.7314453125
52.882824 3196.603271484375
54.27636 3062.576416015625
62.964199 6964.9677734375
63.944725 22855.708984375
78.941261 459038.75
78.958855 3572.82568359375
78.96817 3551.9306640625
81.967178 3013.77294921875
89.836006 3880.827392578125
94.918373 9026.310546875
94.936165 40773.3359375
106.644569 3529.7578125
107.403091 3863.849609375
108.868835 3108.641845703125
109.003441 3187.76708984375
110.913368 112772.203125
111.921188 240659.828125
141.924438 6831.4384765625
141.931763 857676.875
142.931396 3294.34228515625
156.894943 3701.9736328125
156.946457 6378.0576171875
156.955215 1094132.625
167.798874 3430.960693359375
END IONS


BEGIN IONS
NAME=Triclosan
INCHIAUX=
MSLEVEL=MS2
INSTRUMENT_TYPE=Orbitrap
SOURCE_INSTRUMENT=
IONMODE=Negative
Collision energy=
FORMULA=C12H7Cl3O2
EXACTMASS=0.0
PEPMASS=286.9439
ADDUCT=M-H
51.885605 477.20782470703125
51.946533 464.98516845703125
59.511864 462.4769287109375
60.820999 474.3775939941406
64.824623 469.05419921875
67.624748 526.66796875
75.466286 570.2815551757812
```

```
107.817879 466.8296813964844
117.278824 478.0457763671875
131.659088 483.89599609375
166.052811 471.316650390625
171.381882 491.8648681640625
201.071136 733.9882202148438
202.078796 17727.11328125
203.081589 2410.480224609375
203.086945 1405.740478515625
204.386383 531.3063354492188
216.956146 479.0245361328125
273.418945 494.2069396972656
282.8815 525.0193481445312
END IONS


BEGIN IONS
NAME=Zolpidem carboxylic acid
INCHIAUX=
MSLEVEL=MS2
INSTRUMENT_TYPE=Orbitrap
SOURCE_INSTRUMENT=
IONMODE=Negative
COLLISION_ENERGY=
FORMULA=C19H19N3O3
EXACTMASS=337.142641
PEPMASS=336.1354
ADDUCT=M-H
61.331093 4246.23779296875
61.98843 5969.1865234375
63.835423 4519.46630859375
68.628334 3843.668701171875
69.625206 3870.164306640625
72.045372 11772.4189453125
107.061447 10049.9169921875
113.109245 4154.2294921875
202.078796 145611.890625
203.081818 20356.62109375
207.092545 47244.890625
219.093201 26535.455078125
221.108582 38355.59375
235.124023 15695.3515625
247.087616 94493.6875
248.095428 17664.849609375
292.145508 848679.375
304.867584 4420.36669921875
336.135376 221814.34375
END IONS
```
